# Lymphocystis Disease Virus (*Iridoviridae*) Enters Flounder (*Paralichthys olivaceus*) Gill Cells via a Caveolae-Mediated Endocytosis Mechanism Facilitated by Viral Receptors

**DOI:** 10.3390/ijms21134722

**Published:** 2020-07-02

**Authors:** Xiuzhen Sheng, Ying Zhong, Jing Zeng, Xiaoqian Tang, Jing Xing, Heng Chi, Wenbin Zhan

**Affiliations:** 1 Laboratory of Pathology and Immunology of Aquatic Animals, KLMME, Ocean University of China, Qingdao 266003, China; xzsheng@ouc.edu.cn (X.S.); yzhong@shou.edu.cn (Y.Z.); cj7011@stu.ouc.edu.cn (J.Z.); tangxq@ouc.edu.cn (X.T.); xingjing@ouc.edu.cn (J.X.); chiheng@ouc.edu.cn (H.C.); 2 Function Laboratory for Marine Fisheries Science and Food Production Processes, Qingdao National Laboratory for Marine Science and Technology, Qingdao 266071, China

**Keywords:** flounder gill cells, viral receptor, voltage-dependent anion channel protein 2, receptor of activated protein C kinase 1

## Abstract

In previous research, voltage-dependent anion channel protein 2 (VDAC2) and the receptor of activated protein C kinase 1 (RACK1) in flounder (*Paralichthys olivaceus*) were confirmed as functional receptors for lymphocystis disease virus (LCDV) entry; however, the underlying mechanism of VDAC2- and RACK1-mediated LCDV entry remains unclear. In this study, we elucidated the endocytosis pathway of LCDV entry into flounder gill (FG) cells by treatment with specific inhibitory agents, siRNAs, and co-localization analysis. LCDV entry was significantly inhibited by the disruption of caveolae-mediated endocytosis, dynamin, and microtubules, and the knockdown of caveoline-1 and dynamin expression, but was not inhibited by the disruption of clathrin-mediated endocytosis, micropinocytosis, or low-pH conditions. The disruption of caveolae-mediated and clathrin-mediated endocytosis was verified by the internalization of cholera toxin subunit B (CTB) and transferrin, respectively. Confocal immunofluorescence assay demonstrated that LCDV was co-localized with VDAC2 and RACK1, CTB was co-localized with VDAC2 and RACK1 and partially with LCDV, but transferrin was not co-localized with LCDV, VDAC2, or RACK1, indicating that LCDV utilized the same pathway as CTB, i.e., caveolae-mediated endocytosis. This was different from the pathway of transferrin, which used clathrin-mediated endocytosis. Furthermore, caveolin-1 was co-localized with LCDV, VDAC2, and RACK1, suggesting that caveolin-1 was involved in LCDV entry. These results revealed for the first time that LCDV entered into FG cells via caveolae-mediated endocytosis facilitated by VDAC2 and RACK1 receptors, relying on dynamin and microtubules in a pH-independent manner, which provided new insight into the molecular mechanisms of LCDV entry and potential for the development of antiviral agents, expanding our understanding of iridovirus infection.

## 1. Introduction

Lymphocystis disease virus (LCDV), an enveloped DNA virus belonging to the genus *Lymphocystivirus* within the family *Iridoviridae* [1,2], is the causative agent of lymphocystis disease, which infects more than 140 wild and cultured species of marine, brackish, and freshwater fish worldwide [3,4]. Although the disease rarely causes death, it may lead to secondary infection by other microorganisms, resulting in high mortalities [5,6]. The antigenicity, biological and epidemic characteristics, and prevention and treatment of LCDV have been extensively studied [7,8,9,10]. We have identified 27.8 kDa protein as a cellular receptor for LCDV from flounder (*Paralichthys olivaceus*) gill (FG) cells [11], a cell line derived from the gill tissue of a flounder [12]. Moreover, a 32 kDa envelope protein of LCDV was found to function as a viral attachment protein (VAP), and the interaction of the 32 kDa VAP with the 27.8 kDa putative receptor protein initiates LCDV infection in FG cells [13]; monoclonal antibodies (MAbs) against the 32 kDa VAP can effectively neutralize LCDV infection [14]. Recently, voltage-dependent anion channel protein 2 (VDAC2) and the receptor of activated protein C kinase 1 (RACK1) have been identified among the putative 27.8 kDa receptor protein as the functional receptor for LCDV entry [15]. However, the LCDV infection mechanism remains to be elucidated.

Voltage-dependent anion channel protein (VDAC) is abundant in caveolae [16,17,18], along with other proteins and lipids, such as caveolin-1 and cholesterol. In addition, caveolae also contain a large number of signaling molecules, such as G protein, G protein-coupled receptors, and tyrosine kinase receptors, and downstream signaling molecules, e.g., protein kinase A (PKA) and protein kinase C (PKC) [19]. RACK1 functions as a scaffold protein involved in regulation of PKA and PKC proteins [19,20], while PKC is an important regulator of caveolae-mediated endocytosis [21]. Cell membrane VDAC has also been reported to act as a scaffold protein [18]. Although VDAC2 and RACK1 are functional receptors for LCDV, infection by LCDV can be blocked by pre-incubation with anti-VDAC2 and anti-RACK1 antibodies or knockdown of VDAC2 and RACK1 expression through short interfering RNAs (siRNAs), and VDAC2/RACK1 expression on LCDV-nonpermissive epithelial papillosum cell (EPC) conferred susceptibility to LCDV infection [15]. Nonetheless, the underlying mechanism of VDAC2- and RACK1-mediated LCDV entry remains unclear, and further studies focusing on the entry pathway of LCDV facilitated by VDAC2 and RACK1 receptors are required.

Virus-entry pathways are largely defined by interactions between virus particles and their receptors at the cell surface [22]; elucidating this complex interaction is necessary for a full understanding of how viruses invade their hosts [23]. There are two primary routes by which enveloped viruses enter host cells: membrane fusion and endocytosis. The main viral endocytosis pathways are clathrin-mediated endocytosis, caveolae-mediated endocytosis, macropinocytosis, and phagocytosis [21]. The type of endocytosis is not only dependent on the characteristics of the virion itself but is also related to host cytokines, signaling pathways, and receptor proteins [24]. The most commonly used endocytosis pathway is clathrin-mediated endocytosis, which is a continuous process characterized by rapidity and efficiency involving the formation, assembly, and budding of clathrin-coated pits (CCPs) [25]. During the formation of CCPs, clathrin interacts with a series of signaling molecules, including Eps15, adaptor protein AP12, and dynamin GTPase [26,27]. CCPs are then transferred to acidic endosomes, lysosomes, and Golgi apparatus [25]. Endocytic vesicles provide an acidic environment that can help virus uncoating and genome release; therefore, clathrin-mediated endocytosis is sensitive to pH change [28,29]. Another commonly used entry pathway is caveolae-mediated endocytosis, which is a slower process than clathrin-mediated endocytosis. In caveolae-mediated endocytosis, viruses first bind to regions of sphingolipids and cholesterol on the cell membrane known as caveolae, developed from lipid rafts [30,31,32], so this pathway is generally dependent on membrane cholesterol [19]. Caveolae are flask-shaped invaginations of the plasma membrane that are widely distributed and play important roles in signal transduction [19,33]. Caveolin-1 is a marker protein of caveolae formation and also a scaffold protein and regulator of many signaling molecules [34]. Dynamin participates in the formation and scission of CCPs or caveolae, playing important roles in both caveolae-mediated and clathrin-mediated endocytosis [29]. Microtubules serve as tracks for vesicles moving along the membrane system [35,36]. Macropinocytosis is associated with plasma membrane ruffling induced by the activation of actin or microfilaments connected to the plasma membrane [37]. Through this pathway, the virus first activates signaling molecules such as Rac GTPases, Na^+^/H^+^ exchangers, PKC, and phosphoino-sitide 3-kinase (PI3K) [37], which trigger actin remodeling, resulting in membrane folds and bubbles extending from the cell surface. These membrane ruffles fold or fall down on their own to surround the extracellular virus, allowing virus particles to internalize and penetrate into the cytoplasm [38]. Additionally, viruses can use other endocytosis mechanisms to enter host cells, but there have been limited studies on the molecular mechanisms and receptors involved [21,39,40]. Cholera toxin subunit B (CTB) and transferrin are often used as indicators of caveolae- and clathrin-mediated endocytosis, respectively, because CTB is known to enter cells via caveolae-mediated endocytosis, whereas transferrin enters through clathrin-mediated endocytosis [41,42].

Members of the family *Iridoviridae* (iridoviruses) can infect invertebrates and poikilothermic vertebrates, such as insects, fish, amphibians, and reptiles [43,44]. Viruses in the genera, *Lymphocystivirus*, *Megalocytivirus*, and *Ranavirus*, are capable of infecting teleost fish. Infectious spleen and kidney necrosis virus (ISKNV) of the genus *Megalocytivirus* enters host cells via caveolae-dependent endocytosis [35], whereas the Singapore grouper iridovirus (SGIV) of the genus *Ranavirus* enters host cells via clathrin-mediated endocytosis and macropinocytosis pathways in a pH-dependent manner [45]. In the present study, we explored the entry pathway of LCDV into FG cells through interaction with VDAC2 and RACK1 receptor proteins by treating cells with specific inhibitory agents, siRNA, and co-localization analysis. We found that caveolae-mediated endocytosis was involved in LCDV entry. These results not only contribute to elucidating the cellular entry mechanisms of LCDV in fish but also expand our understanding of iridovirus infection.

## 2. Results

### 2.1. Specificity of Mouse Anti-Clathrin, Anti-Caveolin, and Anti-Dynamin Polyclonal Antibodies 

**Figure 1:**
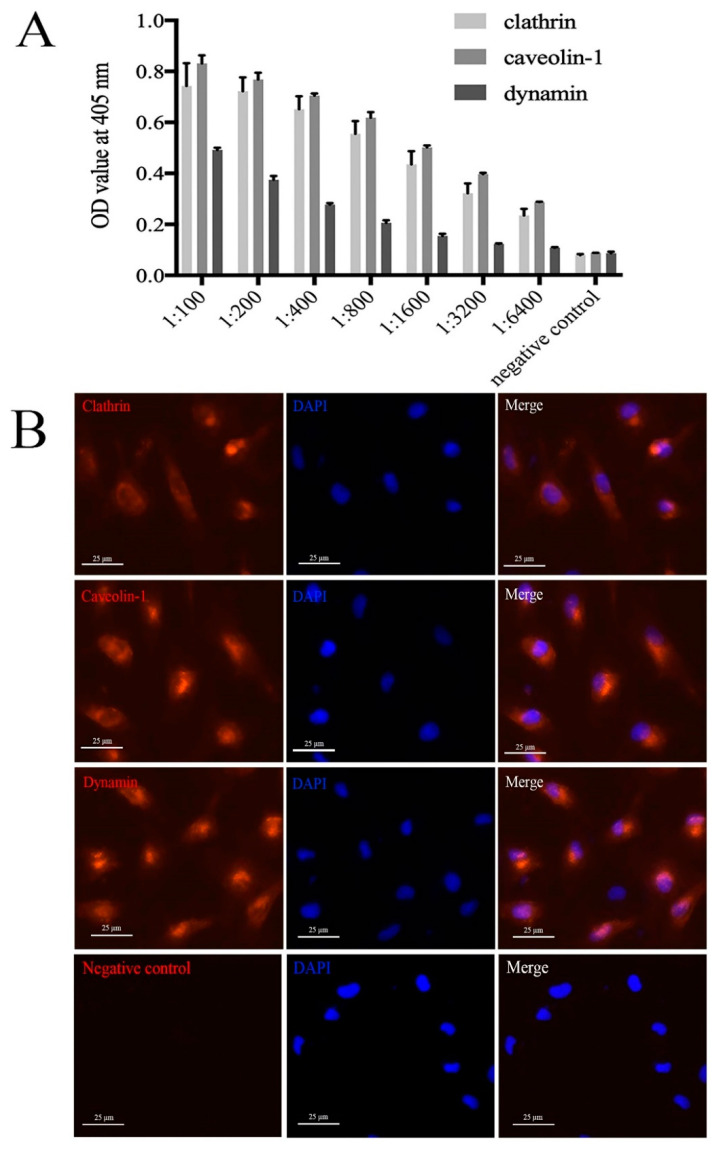
Specificity analysis of mouse anti-clathrin, anti-caveolin-1, and anti-dynamin polyclonal antibodies. (**A**) The titer of mouse anti-clathrin, anti-caveolin-1, and anti-dynamin polyclonal antibodies analyzed by ELISA; error bars represent standard deviations (SD, *n* = 3). (**B**) The indirect immunofluorescence assay (IFA) of mouse anti-clathrin, anti-caveolin-1, and anti-dynamin polyclonal antibodies with FG cells; bars = 25 μm. Mouse pre-immune serum was used as a negative control.

Keyhole limpet hemocyanin (KLH)-conjugated polypeptides of clathrin, caveolin, and dynamin were used to immunize mice, and mouse anti-clathrin, anti-caveolin, and anti-dynamin polyclonal antibodies were produced. The enzyme-linked immunosorbent assay (ELISA) results indicate that the optical density (OD) values of mouse anti-clathrin, anti-caveolin, and anti-dynamin polyclonal antibodies are greatly higher than those of mouse pre-immune serum negative controls; the titer of mouse anti-dynamin polyclonal antibody was slightly lower than those of anti-clathrin and anti-caveolin Figure 1A. Fluorescence-activated cell sorting (FACS) indicated 88.7%, 81.4%, and 81.1% clathrin-, caveolin-, and dynamin-positive cells, respectively Figure S1. Indirect immunofluorescence assay (IFA) showed positive red signals in FG cells, but no positive signals in negative controls, suggesting the mouse anti-clathrin, anti-caveolin, and anti-dynamin polyclonal antibodies could recognize the corresponding proteins on FG cells Figure 1B. All these results indicate that the mouse anti-clathrin, anti-caveolin, and anti-dynamin polyclonal antibodies had good specificity and could be used for the following experiments. 

### 2.2. LCDV Entry Requires Membrane Cholesterol 

**Figure 2:**
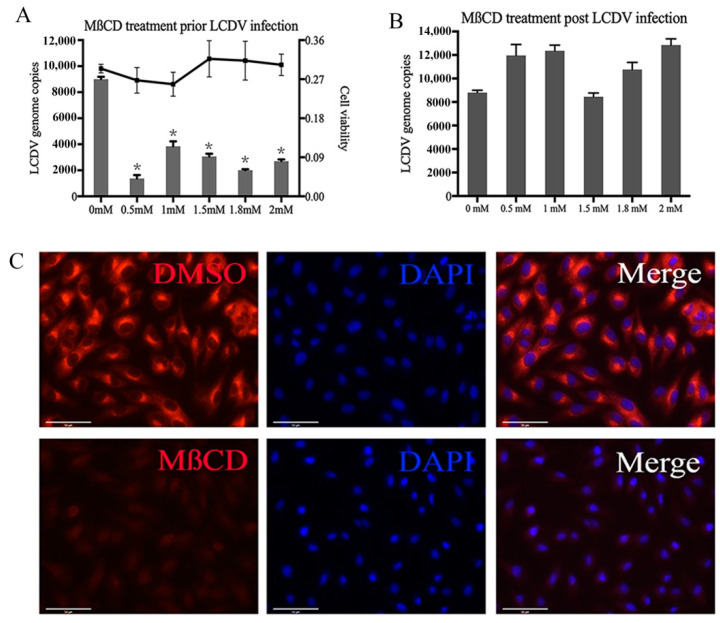
Cholesterol is required for LCDV entry. (**A**) FG cells were pre-incubated with MβCD at 22 °C for 1 h, then incubated with LCDV for another 1 h in the continued presence of reagent. LCDV copy numbers in FG cells were detected by qPCR; the histogram represents virus copy numbers and the line graph represents cell viability. (**B**) FG cells were infected with LCDV for 1 h and then treated with different concentrations of MβCD at 22 °C for 1 h. LCDV copy numbers were detected by qPCR. (**C**) FG cells were treated with 1.5 mM MβCD and then incubated with Alexa fluor 647 conjugated CTB. the fluorescence intensity of CTB in FG cells was observed using fluorescence microscopy; bars = 50 μm. FG cells treated with DMSO instead of MβCD prior to and post LCDV infection served as negative controls. Error bars (**A**,**B**) represent standard deviations (SD, *n* = 3). Asterisks denote significant differences compared with negative controls (*p* < 0.05, one-way ANOVA).

To elucidate whether LCDV entry into FG cells depends on cellular cholesterol, we treated cells with different concentrations of Methyl-β-cyclodextrin (MβCD) to eliminate cholesterol from cell membranes and destroy caveolae-mediated endocytosis both prior to and post LCDV infection. When FG cells were pre-incubated with MβCD for 1 h and then infected with LCDV, the percentage of LCDV-positive cells and virus copy numbers were significantly decreased (*p* < 0.05) (Figure 2A, Figure S2A), but cell viability was not obviously affected by MβCD treatment (Figure 2A). However, when FG cells were treated with MβCD post LCDV entry, the percentage of LCDV-positive cells or virus copy numbers were not obviously decreased regardless of the concentration of MβCD (Figure 2B, Figure S2B). These results indicate that cellular cholesterol was needed for LCDV entry into FG cells, but after virus entry, LCDV replication was not affected by cellular cholesterol.

To verify the disruption of cellular cholesterol, we analyzed the internalization of CTB. The percentage of CTB-positive FG cells was greatly reduced (Figure S2C) and the fluorescence intensity was obviously weakened compared with those of the negative control (Figure 2C), suggesting that cellular cholesterol was eliminated by MβCD. 

### 2.3. LCDV Entry into FG Cells Depends on Dynamin and Microtubules 

**Figure 3:**
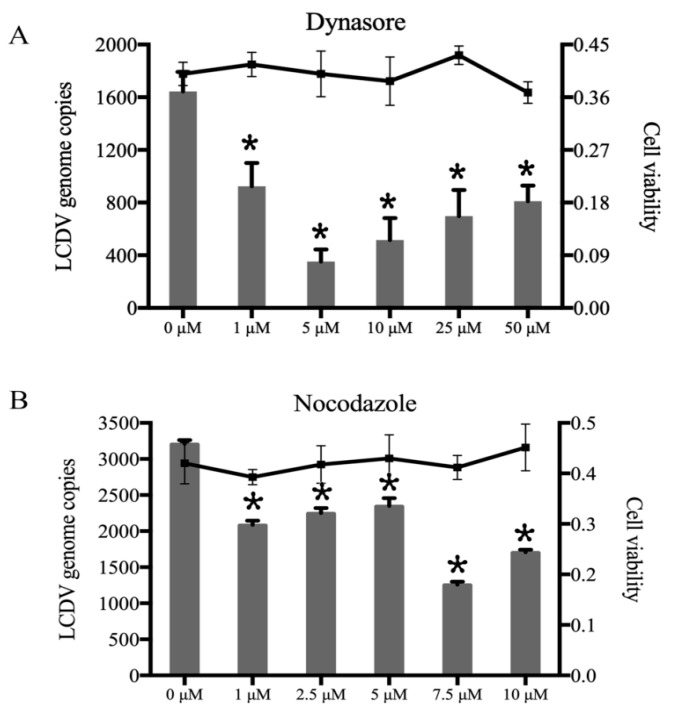
LCDV entry depends on dynamin and microtubules. (**A**) FG cells were pre-incubated with dynasore, and then infected with LCDV. LCDV copy numbers were detected by qPCR; the histogram represents virus copy numbers and the line graph represents cell viability. (**B**) FG cells were pre-incubated with nocodazole and then infected with LCDV. LCDV copy numbers were detected by qPCR; the histogram represents virus copy numbers and the line graph represents cell viability. FG cells pre-incubated with DMSO instead of dynasore or nocodazole served as negative controls. Error bars (**A**,**B**) represent SD (*n* = 3). Asterisks denote significant differences compared with negative controls (*p* < 0.05, one-way ANOVA).

To investigate whether LCDV entry into FG cells is dependent on dynamin and microtubules, we pretreated FG cells with different concentrations of dynasore or nocodazole before LCDV inoculation. FACS showed that, in the presence of greater than 10 µM dynasore or 5 µM nocodazole, the percentage of LCDV-positive cells was decreased as compared with that in negative controls (Figure 3A, Figure S3A); meanwhile, LCDV copy numbers in FG cells were significantly reduced (*p* < 0.05) (Figure 3B, Figure S3B), indicating that dynamin and microtubules were required for LCDV entry. The MTT assay results show that cell viability was not obviously affected by dynasore or nocodazole treatment (Figure 3A,B).

### 2.4. LCDV Entry Is Caveolae-Dependent 

**Figure 4:**
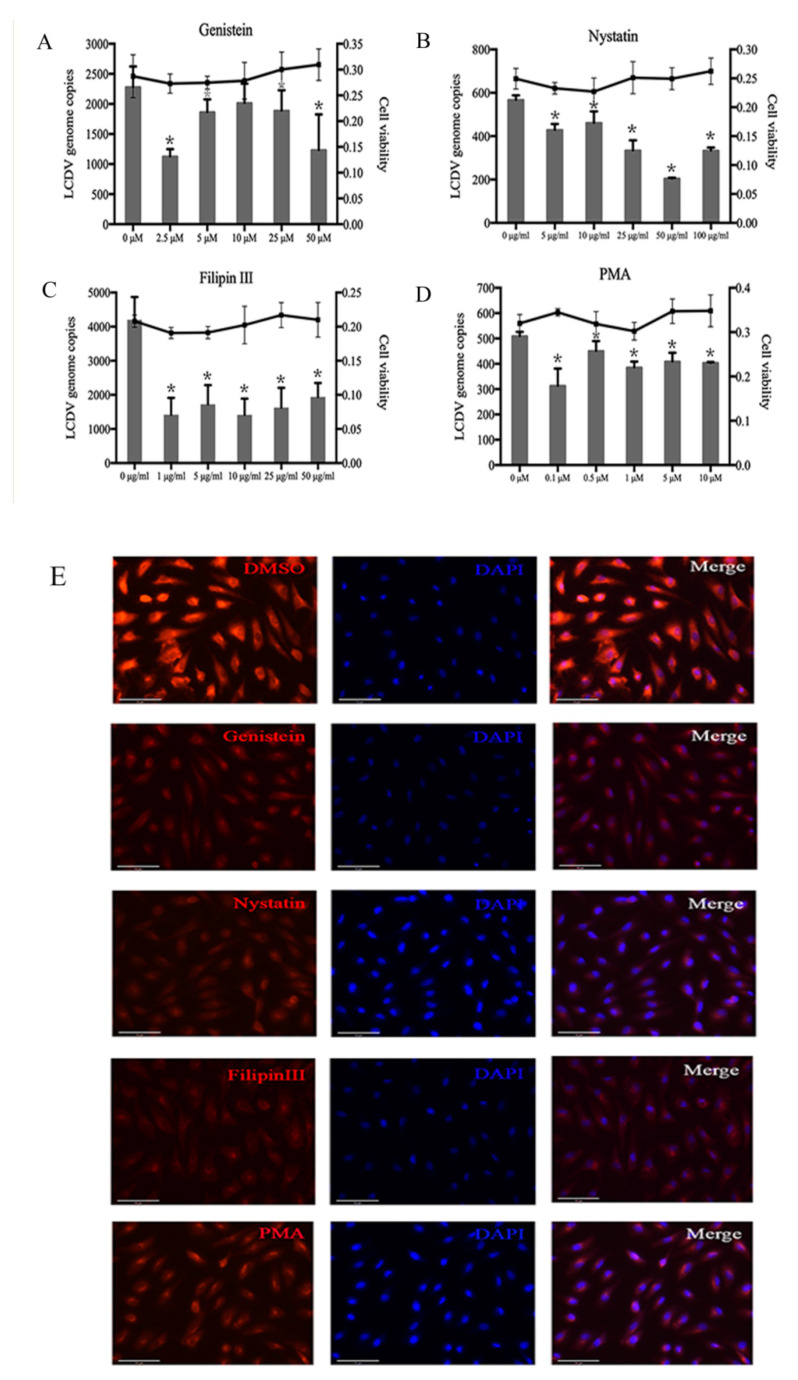
LCDV enters cells via caveola-dependent endocytosis. FG cells were pre-incubated with genistein (**A**), nystatin (**B**), filipin III (**C**), or PMA (**D**), and then infected with LCDV. LCDV copy numbers in FG cells were detected by qPCR; the histogram represents virus copy numbers and the line graph represents cell viability. (**E**) FG cells were treated with 10 μM genistein, 25 μg/mL nystatin, 10 μg/mL filipin III, or 1 μM PMA, then incubated with Alexa fluor 647 conjugated CTB. The fluorescence intensity of CTB in FG cells was observed using fluorescence microscopy; bars = 50 μm. FG cells treated with DMSO instead of genistein, nystatin, filipin III, or PMA served as negative controls. Error bars (**A**–**D**) represent SD (*n* = 3). Asterisks indicate significant differences compared with negative controls (*p* < 0.05, one-way ANOVA).

To disrupt caveolae-mediated endocytosis, we pre-incubated FG cells with different concentrations of the cholesterol-binding reagents nystatin and filipin III, the tyrosine kinase inhibitor genistein, and the PKC activation inhibitor phorbol 12-myristate 13-acetate (PMA) before LCDV infection. The FACS results indicate that the percentage of LCDV-positive cells decreased significantly as compared with negative controls and in the presence of 2.5 µM genistein, 100 µg/mL nystatin, 10 µg/mL filipin III, and 1 µM PMA (Figure S4A–D). The quantitative PCR (qPCR) results demonstrate that LCDV copy numbers were also significantly reduced (*p* < 0.05) (Figure 4A–D). Moreover, we detected the internalization of CTB into FG cells, and the amounts of CTB-positive cells (Figure S4E) and their fluorescence intensity (Figure 4E) were obviously decreased compared with negative controls, indicating that caveolae-mediated endocytosis was disrupted. The MTT assay results reveal that cell viability was not obviously affected by treatment with cholesterol-binding reagents (Figure 4A–D).

### 2.5. LCDV Entry Is pH- and Clathrin-Independent 

**Figure 5:**
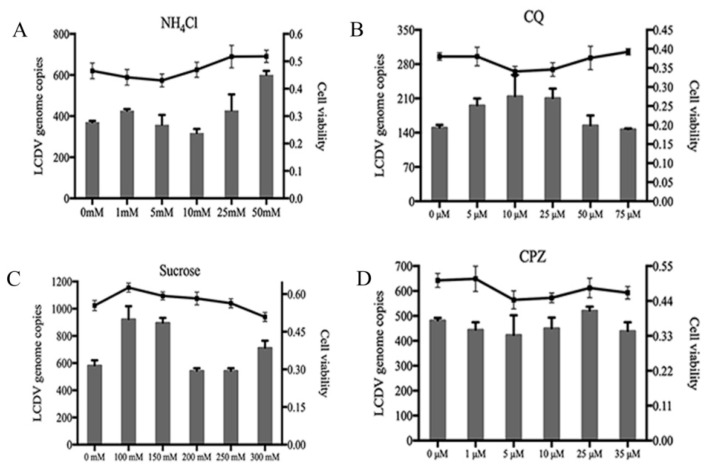

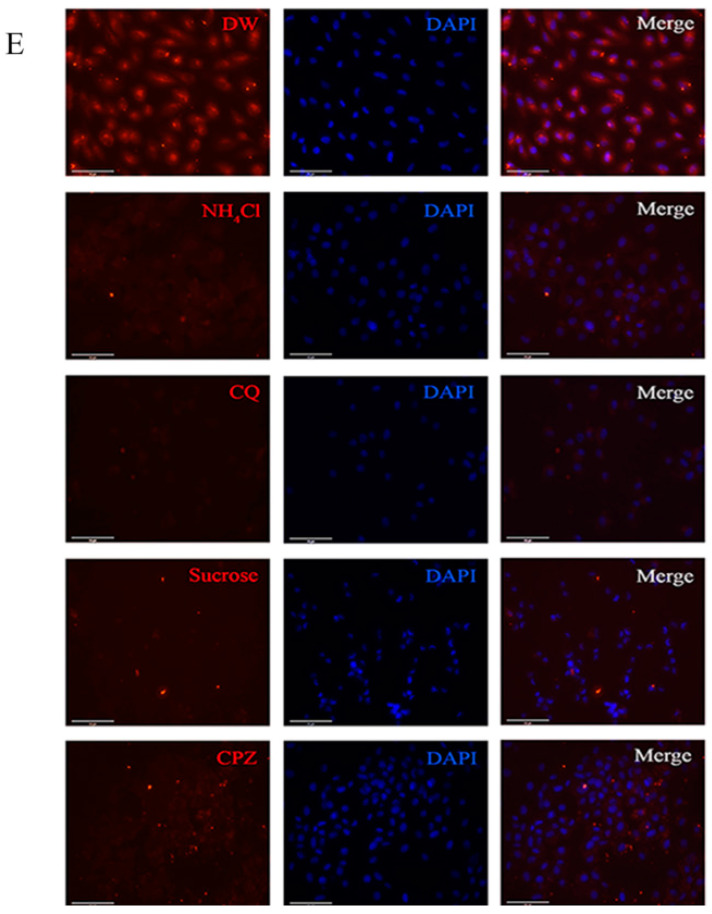
LCDV entry is pH- and clathrin-independent. FG cells were pre-incubated with NH_4_Cl (**A**), CQ (**B**), sucrose (**C**), or CPZ (**D**), and then infected with LCDV. LCDV copy numbers were detected by qPCR; the histogram represents virus copy numbers and the line graph represents cell viability. (**E**) FG cells were treated with 10 mM NH_4_Cl, 25 μM CQ, 200 mM sucrose, or 10 μM CPZ, then incubated with Alexa fluor 647 conjugated transferrin. The fluorescence intensity of transferrin in FG cells was observed using fluorescence microscopy; bars = 50 μm. FG cells treated with distilled water instead of NH_4_Cl, CQ, sucrose or CPZ served as negative controls. Error bars represent SD (*n* = 3).

To verify whether LCDV entry is dependent on cellular pH and clathrin, we disrupted clathrin-mediated endocytosis using sucrose and chlorpromazine (CPZ), and cellular low-pH conditions using chloroquine (CQ) and NH_4_Cl. FG cells were pre-incubated with different concentrations of inhibitory agents before LCDV infection, and the blocking of LCDV entry was detected by FACS and qPCR. No significant changes in the percentages of LCDV-positive cells (Figure S5A,B) or virus copy numbers (Figure 5A,B) were observed in the presence of different concentrations of CQ or NH_4_Cl, indicating that LCDV entry did not correlate with the pH change of endocytic vesicles. Similarly, there were no obvious changes in the percentage of LCDV-positive cells (Figure S5C,D) or virus copy numbers (Figure 5C,D) when FG cells were pre-incubated with different concentrations of sucrose or CPZ, suggesting that clathrin-mediated endocytosis was not involved in LCDV entry.

To verify that the above reagents disrupted clathrin-mediated endocytosis, we the detected internalization of transferrin in FG cells using FACS and IFA, and revealed the decreased amounts of transferrin-positive cells (Figure S5E) and fluorescence intensity (Figure 5E) compared with negative controls. The MTT assay demonstrated that sucrose, CPZ, CQ, or NH_4_Cl treatment had no obvious influence on FG cell viability (Figure 5A–D). These results demonstrate that LCDV entry into FG cells was independent of pH and clathrin-mediated endocytosis. 

### 2.6. LCDV Entry Is Macropinocytosis-Independent

**Figure 6:**
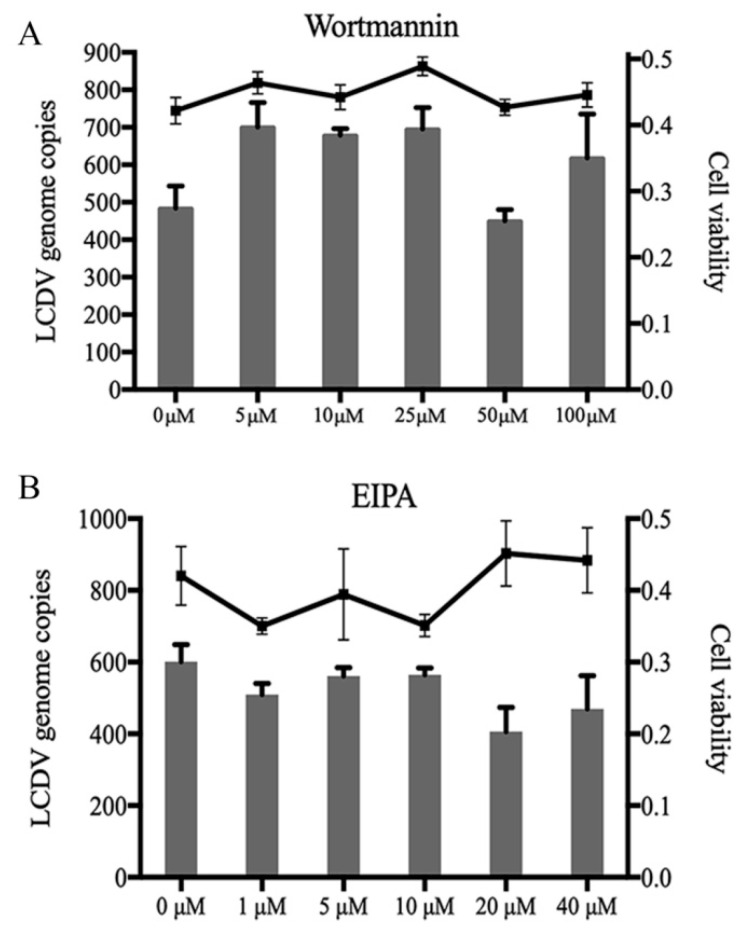
LCDV entry is micropinocytosis independent. FG cells were pre-incubated with wortmannin (**A**) or EIPA (**B**), LCDV copy numbers in FG cells were detected by qPCR; the histogram represents virus copy numbers and the line graph represents cell viability. FG cells treated with DMSO instead of wortmannin or EIPA served as negative controls. Error bars represent SD (*n* = 3).

To elucidate whether LCDV entry into FG cells is dependent on macropinocytosis, FG cells were pretreated with different concentrations of wortmannin to inhibit PI3K and 5-(*N*-ethyl-*N*-isopropyl) amiloride (EIPA) to block Na^+^/H^+^ exchange before LCDV infection; the inhibition of LCDV entry was determined by FACS and qPCR. No obvious changes in the percentage of LCDV-positive cells (Figure S6A,B) or virus copy numbers were observed regardless of wortmannin or EIPA concentration (Figure 6A,B). Cell viability was not affected by reagent treatment as compared with negative controls (Figure 6A,B). These results indicate that LCDV entry was independent of macropinocytosis.

### 2.7. Influence of Clathrin, Caveolin-1, and Dynamin Knockdown in FG Cells on LCDV Infection 

**Figure 7:**
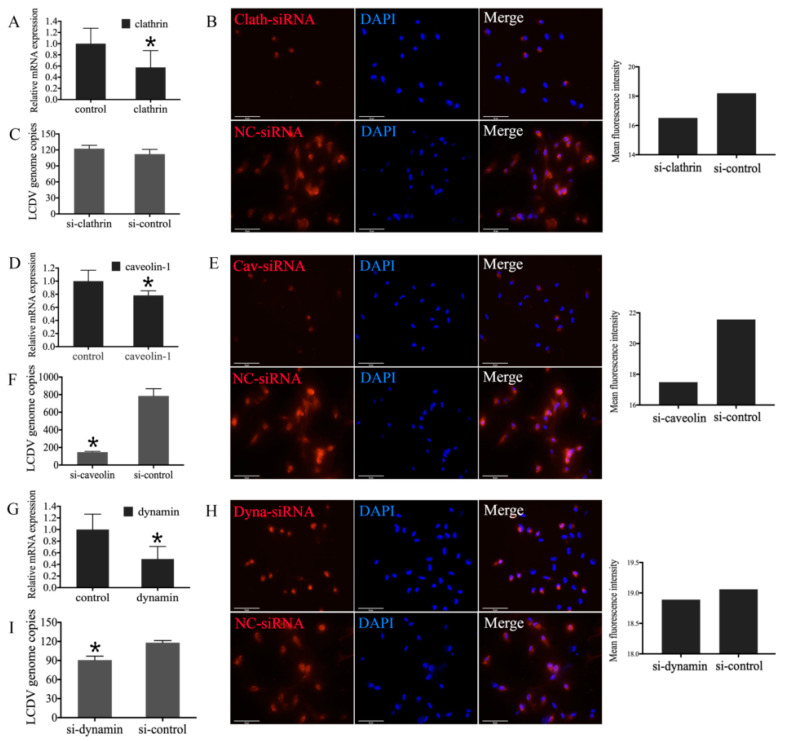
Effect of clathrin, caveolin-1, and dynamin knockdown on the LCDV infection of FG cells. RNA interference sequences were transfected into FG cells and gene expression of clathrin (**A**), caveolin-1 (**D**), and dynamin (**G**) was detected by qPCR; protein expression of clathrin (**B**), caveolin-1 (**E**), and dynamin (**H**) was detected by IFA, bars = 50 μm, the mean fluorescence intensity of all cells in the field was measured using ImageJ software. RNA interference sequences were transfected into FG cells prior to infection with LCDV, and virus copy numbers in clathrin (**C**), caveolin-1 (**F**), and dynamin (**I**) knockdown FG cells were detected by qPCR. Error bars represent SD (*n* = 3). Asterisks denote significant differences compared with negative controls (*p* < 0.05, one-way ANOVA).

To further elucidate the entry pathway of LCDV into FG cells, siRNAs targeting clathrin, caveolin-1, and dynamin 2 (clathrin-siRNA, caveolin-siRNA, and dynamin-siRNA) were transfected into FG cells, and mRNA levels were monitored by qRT-PCR. In comparison with negative controls transfected with a non-silencing control siRNA (NC-siRNA), clathrin, caveolin, and dynamin gene expression in knockdown FG cells was decreased by 0.57-, 0.78-, and 0.49-fold, respectively (*p* < 0.05) (Figure 7A,D,G). As measured using ImageJ software (a free public domain Java image processing program)(Wayne Rasband, National Institutes of Health, Bethesda, MD, USA), the mean fluorescence intensity of clathrin (16.51 versus 18.2), caveolin (17.49 versus 21.57), and dynamin (18.89 versus 19.06) in FG cells was also obviously weakened as compared with the negative controls, which indicated that expression of clathrin, caveolin and dynamin was down-regulated. Here, the results of one group figure for each specific siRNA was provided as an example (Figure 7B,E,H).

To elucidate the effects of clathrin, caveolin, and dynamin knockdown on LCDV infection, the siRNA-transfected FG cells were further infected with LCDV. The qPCR results reveal that LCDV copy numbers in FG cells transfected with caveolin-siRNA or dynamin-siRNA were significantly reduced (*p* < 0.05) (Figure 7F,I), whereas no obvious changes were observed in FG cells transfected with clathrin-siRNA as compared with negative controls (Figure 7C). This indicated that LCDV entry into FG cells was dependent on caveolae-mediated endocytosis and dynamin, but independent of clathrin-mediated endocytosis.

### 2.8. Co-Localization of LCDV with RACK1 and VDAC2

**Figure 8:**
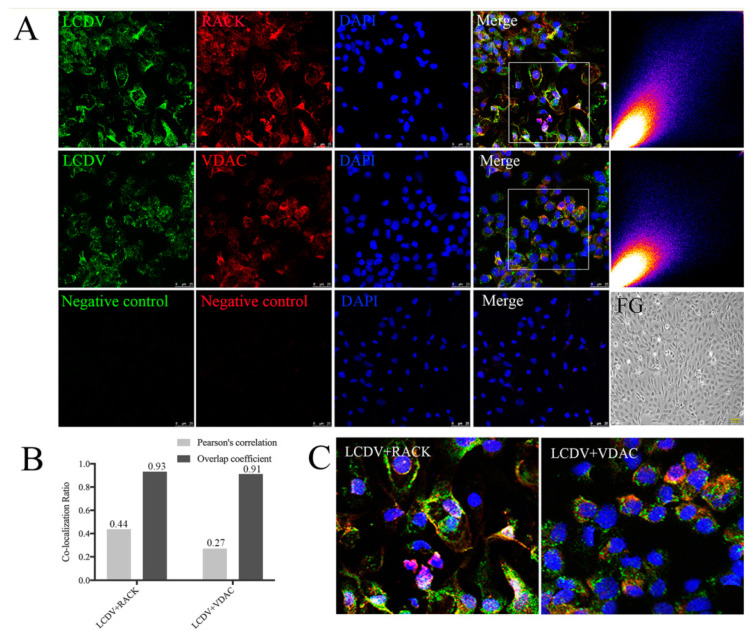
Co-localization of LCDV with VDAC2 and RACK1. (**A**) The co-localization of LCDV with VDAC2 and RACK1 analyzed by confocal microscopy; scatter plots represent co-localization analysis of the merged images using ImageJ software, the diagonal shape of the scattered blots indicated a high level of co-localization, bars = 25 μm. Uninfected FG cells were shown as a control, bars = 100 μm. (**B**) Pearson’s correlation and overlap coefficients of the merged images analyzed by ImageJ software. (**C**) Higher magnification on framed areas of merged figures in (**A**). Rabbit pre-immune serum paired with mouse pre-immune serum instead of primary antibodies served as a negative control. The virus inoculation was conducted at 22 °C for 2 h.

To clarify that LCDV entry into FG cells is mediated by the cellular receptors RACK1 and VDAC2, we determined the co-localization of LCDV with VDAC2 and RACK1 using confocal microscopy. LCDV-positive green signals were present on FG cell membranes and cytoplasm at 2 h post-infection, accompanied by widely distributed red signals representing VDAC2 and RACK1; merged images of LCDV and RACK1, or LCDV and VDAC2, exhibited many yellow co-localization signals in the cell membrane and cytoplasm (Figure 8A,C). We quantified the merged images using ImageJ software, the co-localization level of merged images was quantified by scattered blots, the diagonal shape of the scattered blots indicated a high level of co-localization, the more the diagonal shape gathers, the higher the degree of co-localization was, while distinct bifurcations indicated no co-localization existing. Scatter plots of LCDV with VDAC2 or LCDV with RACK1 assumed a diagonal shape, indicating a high level of co-localization (Figure 8A). Meanwhile, the values of Pearson’s correlation and overlap coefficients indicate higher co-localization between LCDV and RACK1 as compared with LCDV and VDAC2 (Pearson’s correlation of LCDV and RACK1 versus LCDV and VDAC2: 0.44 versus 0.27; Overlap coefficient of LCDV and RACK1 versus LCDV and VDAC2: 0.93 versus 0.91) (Figure 8B). These results reveal that LCDV was co-localized with the two receptor proteins.

### 2.9. Co-Localization of CTB with LCDV, VDAC2, and RACK1 

**Figure 9:**
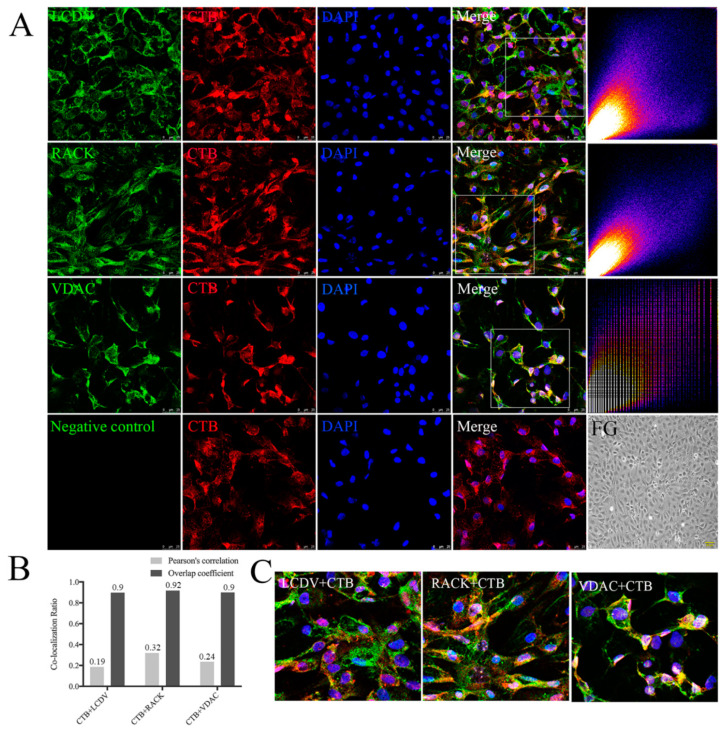
Co-localization of LCDV, VDAC2, and RACK1 with CTB. (**A**) Co-localization of LCDV, VDAC2, and RACK1 with CTB was analyzed by confocal microscopy; scatter plots represent co-localization analysis of the merged images using ImageJ software, the diagonal shape of the scattered blots indicated a high level of co-localization, bars = 25 μm. Uninfected FG cells were shown as a control, bars = 100 μm. (**B**) Pearson’s correlation and overlap coefficients of the merged images analyzed by ImageJ software. (**C**) Higher magnification on framed areas of merged figures in (**A**). Rabbit pre-immune serum instead of primary antibody served as a negative control. LCDV and CTB were inoculated to FG cells at 22 °C for 2 h.

CTB was used as an indicator of caveolae-mediated endocytosis to determine whether LCDV used the same entry pathway as CTB. The confocal microscopy of CTB with LCDV, VDAC2, or RACK1, separately revealed CTB-positive red signals distributed throughout the cells at 2 h post-incubation. LCDV-, VDAC2-, and RACK1-positive green signals were also widely distributed at 2 h post-infection; the merging of CTB images with LCDV, VDAC2, and RACK1 images revealed many yellow co-localization signals in the cell membrane and cytoplasm (Figure 9A,C). The merged images were analyzed using ImageJ software, scatter plots of CTB with VDAC2 or RACK1 presented a diagonal shape, suggesting high co-localization; scatter plots of LCDV with CTB exhibited slight bifurcation, suggesting a lower degree of co-localization than that of CTB with RACK1 and VDAC2 (Figure 9A). The values of Pearson’s correlation and overlap coefficients supported co-localization between CTB and LCDV (0.19 and 0.9), VDAC2 (0.24 and 0.9), and RACK1 (0.32 and 0.92) (Figure 9B). All of these results indicate that CTB was co-localized with LCDV, VDAC2, and RACK1, and that LCDV might adopt the same entry pathway as CTB.

### 2.10. LCDV, VDAC2, and RACK1 Are Not Co-Localized with Transferrin 

**Figure 10:**
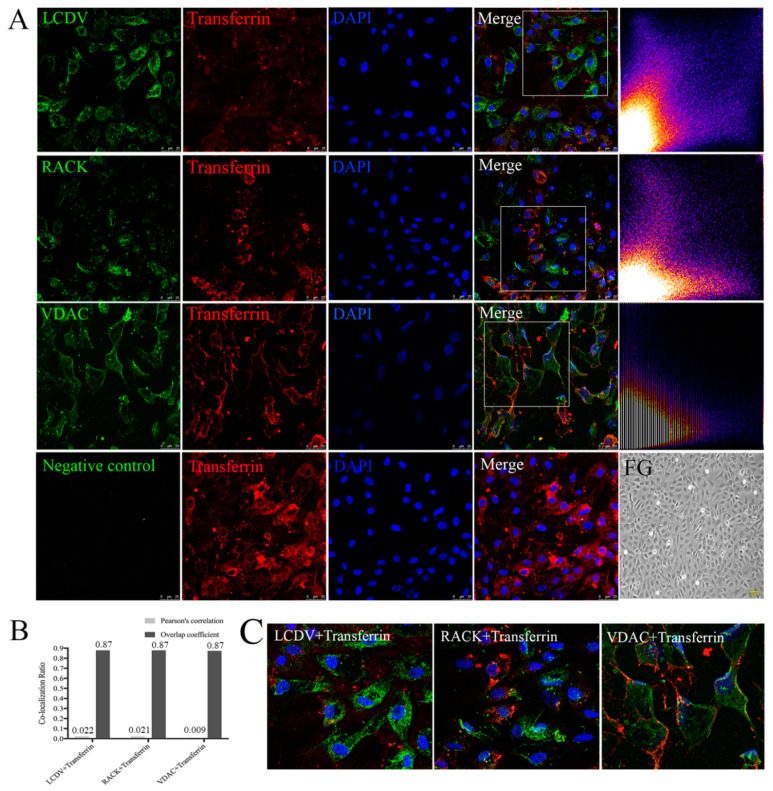
LCDV, VDAC2, and RACK1 do not co-localize with transferrin on FG cells. (**A**) The co-localization of LCDV, VDAC2, and RACK1 with transferrin analyzed by confocal microscopy; scatter plots represent the co-localization analysis of the merged images using ImageJ software, distinct bifurcations of the scattered blots indicated no co-localization existing, bars = 25 μm. Uninfected FG cells were shown as a control, bars = 100 μm. (**B**) Pearson’s correlation and overlap coefficients of the merged images analyzed by ImageJ software. (**C**) Higher magnification on framed areas of merged figures in (**A**). Rabbit pre-immune serum instead of primary antibody served as a negative control. LCDV and transferrin were inoculated to FG cells at 22 °C for 2 h.

To examine differences in entry pathways between LCDV and transferrin, we detected the co-localization of transferrin with LCDV, VDAC2, and RACK1, separately, using confocal microscopy. Transferrin-positive red signals appeared in FG cell membranes and cytoplasm at 2 h post-incubation; LCDV-, VDAC2-, and RACK1-positive green signals were also observed, but no obvious yellow co-localization signals were present in the merged images (Figure 10A,C). Merged images of transferrin with LCDV, VDAC2, and RACK1 were analyzed using ImageJ software, and scatter plots showed distinct bifurcations, indicating that no co-localization existed (Figure 10A). Moreover, Pearson’s correlation and Overlap coefficients of transferrin with LCDV (0.022, and 0.87), VDAC2 (0.009, and 0.87), and RACK1 (0.021, and 0.87) (Figure 10B) were significantly lower than those of CTB with LCDV, VDAC2, and RACK1 (Figure 9B), suggesting that LCDV, VDAC2, and RACK1 did not co-localize with transferrin, and that LCDV might adopt a different entry pathway from transferrin.

### 2.11. Co-Localization of LCDV, VDAC2, and RACK1 with Caveolin 

**Figure 11:**
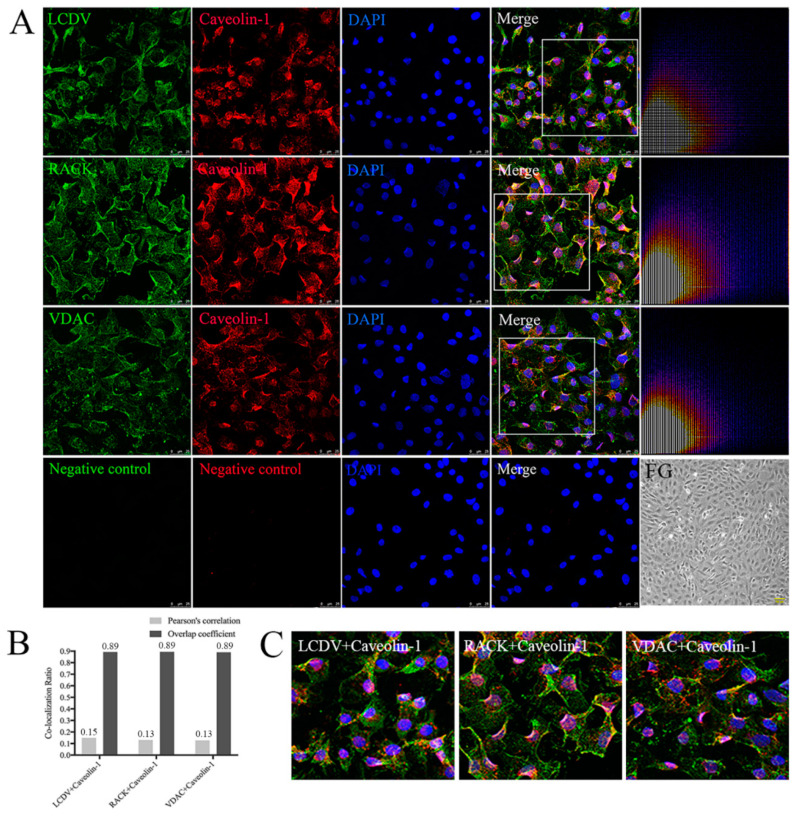
Co-localization of LCDV, VDAC2, and RACK1 with caveolin-1. (**A**) The co-localization of LCDV, VDAC2, and RACK1 with caveolin-1 analyzed by confocal microscopy; scatter plots represent the co-localization analysis of the merged images using ImageJ software, the diagonal shape of the scattered blots indicated a high level of co-localization, bars = 25 μm. Uninfected FG cells were shown as a control, bars = 100 μm. (**B**) Pearson’s correlation and overlap coefficients of the merged images analyzed by ImageJ software. (**C**) Higher magnification on framed areas of merged figures in (**A**). Rabbit pre-immune serum paired with mouse pre-immune serum instead of primary antibodies served as a negative control. The virus inoculation was conducted at 22 °C for 2 h.

Co-localization analysis of LCDV, VDAC2, and RACK1 with caveolin (a marker protein of caveolae-mediated endocytosis) using confocal microscopy revealed extensive LCDV-, VDAC2-, and RACK1-positive green signals in FG cell membranes and cytoplasm, accompanied by widely distributed caveolin-positive red signals at 2 h post LCDV infection; merged images of caveolin with LCDV, VDAC2, and RACK1 exhibited many yellow co-localization signals (Figure 11A,C). We further analyzed the merged images of LCDV, VDAC2, and RACK1 with caveolin using ImageJ software, and no bifurcations were observed in the scatter plots, indicating that co-localization existed (Figure 11A). The values of Pearson’s correlation and overlap coefficients for caveolin with LCDV (0.15 and 0.89), VDAC2 (0.13 and 0.89), and RACK1 (0.13 and 0.89) (Figure 11B) were much higher than those of transferrin with LCDV, VDAC2, and RACK1 (Figure 10B). Therefore, caveolin co-localized with LCDV, VDAC2, and RACK1, and LCDV entry into FG cells was dependent on caveolae, mediated by VDAC2 and RACK1 receptor proteins.

## 3. Discussion

Viruses enter host cells by fusion, permeation, or endocytic vesicle discharge and exit them by budding or membrane disruption [46]. Although some viruses can penetrate directly into the cytoplasm, most viruses are transported across the plasma membrane through an endocytosis pathway and then transferred to other intracellular organs [47]. An understanding of the endocytosis pathway is critical for elucidating viral infection mechanism and developing antiviral agents. Iridoviruses are capable of infecting invertebrates and poikilothermic vertebrates, causing great economic losses in the aquaculture industry and showing a significant threat to global biodiversity [43,44]; however, the molecular mechanism underlying iridovirus entry into cells is not well understood. Comparatively, we know more about the entry pathway of the genus *Ranavirus*. Frog virus 3 (FV3), which is a type species of the genus *Ranavirus* and recognized as a model for iridoviruses, enters into mammalian cells (BHK-21) via the clathrin-mediated endocytosis [48], while tiger frog virus (TFV) enters into HepG2 cells via the caveola-mediated endocytosis pathway in a pH-dependent manner [49], and SGIV enters into the grouper spleen (GS) cells by clathrin-mediated endocytosis and micropinocytosis [45]. Among iridoviruses, viruses in the genus Lymphocystivirus, *Megalocytivirus*, and *Ranavirus* can infect teleost fish. It is found that ISKNV (the genus *Megalocytivirus*) also enters host cells via caveolae-dependent endocytosis [35]. However, no studies of the viral receptor proteins involved in the entry of SGIV and ISKNV have been reported [35,46]. In LCDV infection, we previously observed that the LCDV 32 kDa VAP interacts with VDAC2 and RACK1 receptors to initiate virus infection. VDAC2/RACK1 knockdown through siRNA significantly reduced LCDV copy numbers, whereas VDAC2/RACK1 expression on LCDV-nonpermissive EPC conferred susceptibility to LCDV infection [15]. The LCDV 32 kDa VAP is encoded by the ORF038 gene of LCDV isolated in China (LCDV-C) [14,50], and the LCDV-C ORF038 gene had homologues with genes encoding SGIV VP19 and rana grylio virus envelope protein 2L [50,51]. In the present study, we found that LCDV enters FG cells via caveolae-mediated endocytosis facilitated by VDAC2 and RACK1 receptors, relying on dynamin and microtubules in a pH-independent manner, but clathrin-mediated endocytosis and macropinocytosis are not involved. 

Caveolin and clathrin are specific biomarkers of caveolae-mediated endocytosis and clathrin-mediated endocytosis, respectively [21,34], while dynamin plays an important role in both endocytosis pathways [35]. Clathrin is assembled on the inside face of the plasma membrane to form a CCP [52]. Caveolins, a family of integral membrane proteins, are the structural proteins of caveolae [16], caveolin and dynamin shows a significant colocalization on plasmalemmal caveolae in lung endothelial cells [19]. We determined that the expression levels of caveolae, clathrin, and dynamin in FG cells were similar, using specific anti-caveolae, -clathrin, and -dynamin polyclonal antibodies evaluated by ELISA, IFA, and FACS analysis. Cellular membrane cholesterol is a constituent of caveolae and may be involved in different stages of the virus life cycle [33,53]. To address the role of cellular membrane cholesterol in LCDV infection, we eliminated FG cell cholesterol with MβCD prior to and post LCDV infection, respectively. LCDV entry was only severely inhibited by MβCD treatment before virus infection, suggesting that cholesterol is important for the endocytosis of LCDV, while the subsequent LCDV life cycle, such as internalization or uncoating, might be independent of cholesterol. By contrast, in classic swine fever virus, removal of cell membrane cholesterol post infection significantly inhibits virus egression [53]. Caveolae-mediated endocytosis depends on cholesterol, but cholesterol consumption may cause acute effects and affect caveolae-independent endocytosis indirectly. Cholesterol in cellular membranes is essential for productive infection of African swine fever virus and classical swine fever virus, although they enter host cells via clathrin-mediated endocytosis [54,55]; cholesterol dependence is therefore not equivalent to caveolae dependence. In this study, the disruption of caveolae-mediated endocytosis by the cholesterol-binding reagents nystatin, filipin III, the tyrosine kinase inhibitor genistein, and the PKC activation inhibitor PMA severely impaired LCDV entry. LCDV-positive cells and copy numbers significantly decreased after treatment of reagents in various concentrations. All these results reveal that caveolae-mediated endocytosis depended on cholesterol. However, no clear dose-dependent inhibitory effect was observed in our study, which was not consistent with the results for ISKNV [35]; although we used similar reagent concentrations. This might be because of the difference in cells and virus species, or proportions of cell numbers and reagent concentrations, so more studies are needed. Furthermore, the disruption of caveolae-mediated endocytosis was verified by the internalization of CTB, which enters cells via caveolae-mediated endocytosis [41]. Meanwhile, LCDV copy numbers were significantly reduced in caveolin-1 knockdown FG cells, supporting the concept that LCDV entry into FG cells proceeds via caveolae-mediated endocytosis, as caveolin-1 is important for caveolae formation and scission [56]. However, with little inhibition of caveolin gene transcription, there was significant inhibition of caveolin protein expression, which might be because the reduction of target protein needed more time than its gene, or there may exist an optimal time point that the expression of gene and protein reduced simultaneously. Further research also is required in the future. Dynamin and microtubules play important roles in caveolae-mediated endocytosis, with dynamin participating in the scission of caveolae and microtubules acting as tracks for endocytic vesicle movement [35]. When we disrupted dynamin and microtubules using specific reagents, LCDV entry was significantly inhibited, and LCDV copy numbers were reduced after dynamin expression was knocked down. Similar results were also obtained for enterovirus 71, the virus RNA was significantly blocked in Jurkat T and PSGL-1-L929 cells when disturbing caveolar endocytosis by caveolin-1 siRNA [57]. It is possible for siRNAs to produce differences in the specific silencing of a target gene in different experiments; therefore, a more specific and stable knockout technique, such as the Crispr-Cas system, might be more effective in further research. However, the results in this study reveal that LCDV entry occurs via caveolae-mediated endocytosis and is dependent on dynamin and microtubules.

Clathrin-mediated endocytosis and macropinocytosis are two other important endocytosis pathways for viruses [40,58]. Virus entry via clathrin-mediated endocytosis usually depends on pH [45,55]; however, entry of enterovirus 71, which adopts caveolae-mediated endocytosis, is also dependent on intracellular pH [57]. Both caveolae-mediated and clathrin-mediated endocytosis are dependent on dynamin, while macropinocytosis is independent of dynamin [28,59]. Some viruses may adopt more than one endocytosis pathways, such as influenza A virus, which usually enters cells via clathrin-mediated endocytosis, but in some cases can activate macropinocytosis [60,61]. In the present study, the ability of LCDV to infect FG cells was not affected by disruption of cellular low-pH conditions, clathrin-mediated endocytosis, or macropinocytosis, because the percentages of LCDV positive cells and virus copy numbers were not obviously decreased as compared with negative control, indicating that LCDV entry is pH-, clathrin-, and macropinocytosis-independent. The disruption of clathrin-mediated endocytosis was verified by the internalization of transferrin, which enters cells via clathrin-mediated endocytosis [42]. Besides, LCDV copy numbers were not decreased when clathrin expression in FG cells was knocked down, further indicating that LCDV entry is independent of clathrin-mediated endocytosis. However, some viruses may adopt different endocytosis pathways when entering different types of cells, such as enterovirus 71 (EV71), which enters human rhabdomyosarcoma (RD) cells via clathrin-mediated endocytosis, but activate caveolar endocytosis when infect Jurkat T and mouse L929 cells [57]. Since LCDV could infect other cell types, such as flounder embryo cells [62], understanding whether LCDV adopt the same endocytosis pathway in these cells requires further research.

When invading host cells, viruses first bind to cellular receptor proteins, and the process of entry mediated by receptor proteins is then initiated [23,63]. Viruses may adopt distinct entry pathways due to differences in receptor proteins [57,64,65,66]. For viruses that enter via caveolae-mediated endocytosis, receptor proteins are usually abundant in the caveolae of susceptible cells [67]. VDAC2 and RACK1 are widely distributed in caveolae [16,17,18,19,20,21], and VDAC2 and RACK1 knockdown or blocking inhibits LCDV infection of FG cells [15]. To elucidate the roles of VDAC2 and RACK1 in LCDV entry, we confirmed that LCDV could co-localize with VDAC2 and RACK1, and CTB also co-localized with VDAC2 and RACK1, while LCDV partially co-localized with CTB, indicating that VDAC2 and RACK1 mediate LCDV infection, and CTB might co-localize with VDAC2 and RACK1 during LCDV entry, even though the receptor protein for CTB in caveolae is ganglioside [68]. This might be because CTB assembles with VDAC2 and RACK1 in the caveolae, and LCDV might be in competition with CTB since they all adopt caveolae-mediated endocytosis. However, we detected no co-localization of transferrin with LCDV, VDAC2, or RACK1, suggesting that the LCDV entry pathway is different from that of transferrin, which is dependent on clathrin-mediated endocytosis; therefore, co-localization analysis of LCDV and clathrin was not performed in this study. Caveolin-1 was co-localized with LCDV, VDAC2, and RACK1, and we suggest that VDAC2 and RACK1 might assemble in caveolae to mediate the entry of LCDV via caveolae-mediated endocytosis, since caveolin-1 participates in the formation of caveolae where VDAC2 and RACK1 are widely distributed [16,17,18,19,20,23,34]. There may be differences in the timing and manner in which these two receptors work, and the specific roles of the two receptors in LCDV entry needs to be further clarified. 

**Figure 12:**
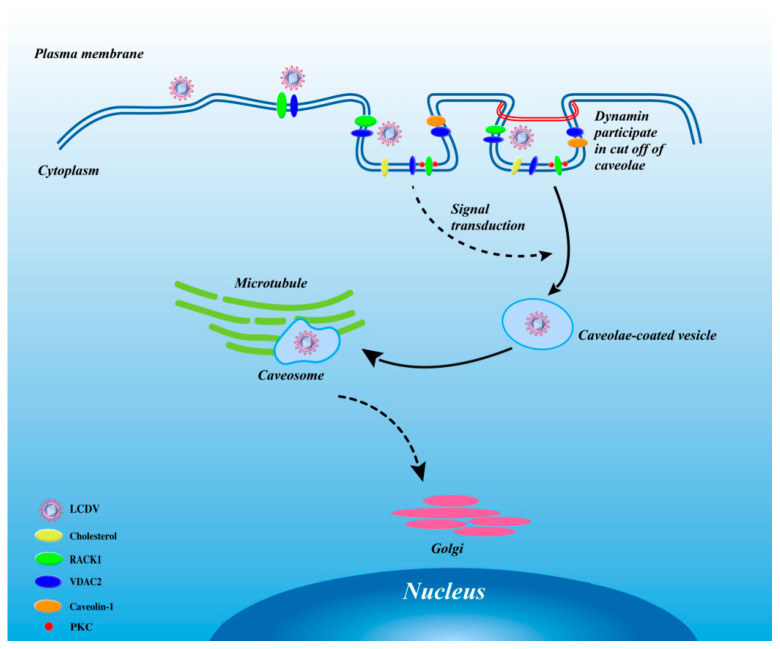
Model of LCDV entry via the caveolae-mediated endocytosis pathway facilitated by viral receptors. Solid arrow represented the LCDV entry steps has been proved in the present study, and dotted arrow represented potential steps basing on other published research.

Together, our results support a model of LCDV entry via the caveolae-mediated endocytosis pathway facilitated by viral receptors VDAC2 and RACK1 (Figure 12). We propose the following model: the virus first interacts with cellular receptors VDAC2 and RACK1 on the FG cell surface and is then internalized into caveolae, which are primarily composed of cholesterol and caveolin-1; virus–receptor interaction activates the caveolae-mediated endocytosis pathway and the virus is further internalized. RACK1 might also function as a scaffolding protein to enrich signaling molecules. PKC and VDAC2 might also participate in the apoptotic signaling pathway during LCDV internalization [19,20,69], but the specific mechanism here needs more studies. Dynamin forms a ring to pinch off the caveolae, and the virus in a caveolae-coated vesicle is transferred into cells, moving along microtubules. Uncoating is initiated and viral nucleic acid is released when the vesicle arrives at its final destination. Since the definition of caveolae-mediated endocytosis has been revised in previous study [70], more studies of the interaction of the two viral receptors with caveolin-1 in LCDV entry are required to further modify the hypothetical model.

In conclusion, we confirmed that LCDV enters FG cells via caveolae-mediated endocytosis facilitated by VDAC2 and RACK1 receptors, relying on dynamin and microtubules in a cholesterol-dependent and pH-independent manner; clathrin-mediated endocytosis and the macropinocytosis pathway are not involved. The LCDV endocytosis pathway is similar to that of ISKNV and TFV, which also use caveolae-mediated endocytosis [35,49], but TFV entry is pH dependent [35]; In contrast, FV3 enters cells via the clathrin-mediated endocytic pathway [48], and SGIV uses clathrin-mediated endocytosis and micropinocytosis [45]. This study of the LCDV entry pathway not only allows for further elucidation of the molecular mechanism of LCDV infection and provides potential for the development of antiviral agents, but also expands our understanding of iridovirus pathogenesis.

## 4. Materials and Methods

### 4.1. Ethics Statement 

The present study was conducted in strict accordance with the recommendations in the Guide for the Use of Experimental Animals of Ocean University of China. The protocols for animal care and handling were approved by the Institutional Animal Care and Use Committee of the Ocean University of China (Permit Number: 20151201, offer date: 2015. 12. 01). All efforts were dedicated to minimizing suffering.

### 4.2. Cells, Virus and Antibodies

FG cells were cultured in Minimal Essential Medium (MEM) (Gibco, CA, USA) supplemented with 10% fetal bovine serum (FBS) (Gibco, Grand Island, CA, USA), 100 IU/mL penicillin and 100 μg/mL streptomycin (Gibco, Grand Island, CA, USA), and cultivated at 22 °C with 2% CO2. FBS was reduced to 2% in maintenance medium post LCDV infection. Virus strain LCDV-HD (GenBank accession number: DQ279090) was purified in previous studies and stored at −80 °C [13,14]. 

Rabbit anti-LCDV 32 kDa VAP polyclonal antibody, mouse anti-32 kDa VAP MAb, rabbit anti-RACK1 polyclonal antibody, and rabbit anti-VDAC2 polyclonal antibody were produced in the authors’ laboratory [13,14,15]. Alexa Fluor 647 conjugated goat anti-mouse or -rabbit Ig, Alexa Fluor 488 conjugated goat anti-mouse or -rabbit Ig, and Alexa Fluor 647 conjugated CTB (recombinant) or transferrin from human serum were purchased from Thermo Fisher (Waltham, MA, USA).

### 4.3. Production and Specificity Analysis of Mouse Anti-Clathrin, Anti-Caveolin, and Anti-Dynamin Polyclonal Antibodies 

Mouse anti-clathrin, anti-caveolin, and anti-dynamin polyclonal antibodies were obtained by immunizing mice with polypeptides designed and synthesized by Genscript (Nanjing, China). Peptide sequences of clathrin, caveolin-1, and dynamin are listed in Table 1. For the first intraperitoneal immunization, 200 mg KLH-conjugated polypeptide diluted in phosphate-buffered saline (PBS) was emulsified with equal volume of complete Freund’s adjuvant (Sigma, St. Louis, MO, USA, 1:1). Two weeks later, a booster immunization was performed by administering 200 mg KLH-conjugated polypeptide emulsified with incomplete Freund’s adjuvant (Sigma, St. Louis, MO, USA, 1:1) through intraperitoneal injection. Mice were thereafter given two booster immunizations with 100 mg KLH-conjugated polypeptide through caudal vein injection at one-week intervals. Three days after the last immunization, blood was obtained and placed at room temperature for 1 h and then at 4 °C overnight before centrifugation at 10,000× *g* for 30 min. The supernatant was collected and stored at −80 °C until use. The specificity of mouse anti-clathrin, anti-caveolin, and anti-dynamin polyclonal antibodies was determined by ELISA, FACS, and IFA. 

For FACS, FG cells grown in 75 cm^2^ culture flasks were digested with trypsin and washed with PBS before fixing with 4% paraformaldehyde for 15 min at 22 °C. Subsequently, cells were incubated with the above mouse polyclonal antibodies separately at 37 °C for 1.5 h and then washed three times with PBS. Cells were further incubated with Alexa Fluor 647 conjugated goat anti-mouse Ig at 37 °C for 45 min and then washed as above. The percentage of clathrin-positive, caveolin-positive, and dynamin-positive FG cells was analyzed by FACS using flow cytometry (Beckman Counter, Fullerton, CA, USA) [71,72]. Mouse pre-immune serum instead of primary antibodies served as negative controls. Three replicates were performed.

For IFA, FG cells were seeded on circular coverslips in 24-well plates and fixed with 4% paraformaldehyde as above. Cells were then washed three times with PBS and incubated with the above mouse polyclonal antibodies separately at 37 °C for 1.5 h. After washing three times with PBS, cells were further incubated with Alexa Fluor 647 conjugated goat anti-mouse Ig at 37 °C for 1 h and washed again. Cell nuclei were visualized using 4,6-diamidino-2-phenylindole (DAPI, Invitrogen, San Diego, CA, USA) and clathrin, caveolin-1, and dynamin were detected by fluorescence microscopy (Olympus, Tokyo, Japan). Mouse pre-immune serum instead of primary antibodies served as negative controls. Three replicates were performed.

### 4.4. Drug Treatment and Cytotoxicity Assay 

The chemical reagents used in this study were purchased from Sigma-Aldrich (St. Louis, MO, USA) and prepared according to [35,45,73]. Ammonium chloride (NH_4_Cl) (1, 5, 10, 25, 50 mM) and CQ (5, 10, 25, 50, 75 µM) diluted in distilled water were used to disrupt the low-pH condition of endosomes and lysosomes in FG cells. MβCD (0.5, 1, 1.5, 1.8, 2 mM) diluted in dimethyl sulfoxide (DMSO) solution was used to disrupt lipid rafts by depleting cholesterol. Dynasore (1, 5, 10, 25, 50 µM) and nocodazole (1, 2.5, 5, 7.5, 10 µM) diluted in DMSO were used to disrupt cellular dynamin and microtubules, respectively. Sucrose (100, 150, 200, 250, 300 mM) and CPZ (1, 5, 10, 25, 35 µM) in distilled water served as inhibitory agents of CCP formation to disrupt clathrin-mediated endocytosis. For the depletion of caveolae-mediated endocytosis, filipin III (1, 5, 10, 25, 50 µg/mL) and nystatin (5, 10, 25, 50, 100 µg/mL) in DMSO were used to bind cholesterol, genistein (2.5, 5, 10, 25, 50 µM) and PMA (0.1, 0.5, 1, 5, 10 µM) in DMSO was used to inhibit tyrosine kinase and PKC, respectively. To inhibit the macropinocytosis pathway, wortmannin (5, 10, 25, 50, 100 µM) as a PI3K inhibitor and EIPA (1, 5, 10, 20, 40 µM) that blocks Na^+^/H^+^ exchange, diluted in DMSO, were used. 

To disrupt the endocytosis pathway for LCDV entry, NH_4_Cl, CQ, MβCD, dynasore, nocodazole, sucrose, CPZ, filipin III, nystatin, genistein, PMA, wortmannin and EIPA at different concentrations were used to treat FG cells prior to LCDV infection. FG cells grown in 25 and 75 cm^2^ culture flasks with 90% confluence were washed three times with PBS and treated with different concentrations of reagents at 22 °C for 1 h. Cells were then inoculated with LCDV at 4 median tissue culture infective dose (TCID_50_/mL) as described previously [11,74] for another 1 h in the continued presence of the reagents at 22 °C with 2% CO2. MβCD with various concentrations was used to treat FG cells at 22 °C for 1 h prior to or post LCDV infection in the continued presence of reagent. Cells were washed once with citrate buffer (40 mM sodium citrate, 10 mM KCl, 135 mM NaCl, pH 3.1) for 50 s to inactivate the virus and three times with PBS to remove residual citrate buffer [35,45], and then cultivated in 2% maintenance medium for 24 h. FG cells treated with corresponding solvents instead of the reagents served as negative controls. Cells grown in 75 cm^2^ culture flasks were digested using trypsin, centrifuged at 800× *g* for 5 min, and then washed three times with PBS. After fixed with 4% paraformaldehyde for 15 min at room temperature and washing three times with PBS, cells were incubated with rabbit anti-LCDV 32 kDa VAP polyclonal antibody (1:500) for 1.5 h, followed by Alexa Fluor 647 conjugated goat anti-rabbit Ig for 45 min at 37 °C. After washing again, the percentage of LCDV-infected FG cells was detected by FACS. FG cells grown in 25 cm^2^ culture flasks were digested and centrifuged, the DNA of these cells was extracted using a TIANamp Marine Animals DNA Kit (Qiagen, Hilden, Germany) following the manufacturer’s instructions, and LCDV copy numbers were detected by qPCR. The qPCR reaction conditions were as follows: 10 min at 95 °C, followed by 45 cycles of 10 s at 95 °C, 10 s at 55 °C, and 20 s at 72 °C. LCDV copy numbers were calculated using a standard curve according to Ct values as described previously [13].

To confirm whether clathrin-mediated endocytosis in FG cells could be disrupted by specific chemical reagents, cells were treated with NH_4_Cl (5 mM), CQ (10 µM), sucrose (150 mM), or CPZ (10 µM) at 22 °C for 1 h, and then incubated with Alexa Fluor 647 conjugated transferrin (10 µg/mL) at 22°C for 1 h. Similarly, FG cells were incubated with Alexa Fluor 647 conjugated CTB (20 µg/mL) after treatment with MβCD (1.5 mM), nystatin (25 µg/mL), genistein (10 µM), or filipin III (10 µg/mL) to disrupt caveolae-mediated endocytosis. After washing cells with citrate buffer and PBS, the percentage of transferrin-positive and CTB-positive cells was detected by FACS. Finally, the internalization of transferrin and CTB to FG cells was observed under a fluorescence microscope (Olympus, Tokyo, Japan). FG cells treated with the corresponding solvent instead of reagents served as negative controls. 

MTT assay was used to detect the influence of chemical reagents on FG cell viability. FG cells were seeded in 96-well plates and grown to a monolayer before incubating with different concentrations of reagents for 2 h. Reagents were then removed and cells were washed with citrate buffer and PBS. Cells were incubated with 50 μL MTT (Sigma, St. Louis, MO, USA) at 22 °C for 4 h. Thereafter, the supernatant was removed and 150 μL DMSO was added and incubated for 10 min. Absorbencies were measured with an automatic ELISA reader (Molecular Devices, Union city, CA, USA) at 490 nm.

### 4.5. RNAi Knockdown of Clathrin, Caveolin-1, and Dynamin 

The clathrin, caveolin-1, and dynamin 2 in FG cells were knocked down by using siRNAs designed by Shanghai GenePharma Company (Shanghai, China). The siRNA sequences targeting clathrin, caveolin-1, and dynamin 2 and the control NC-siRNA are shown in Table 1. FG cells were grown in six-well plates, and siRNAs were separately transfected into FG cells using Lipofectamine 3000 reagent (Invitrogen, Carlsbad, CA, USA) according to the manufacturer’s instructions. Briefly, 5 μL (20 μM) of siRNA and 3.25 μL Lipofectamine 3000 reagent were diluted with 125 μL opti-MEM (Gibco, Grand Island, CA, USA) separately, mixed and incubated for 20 min at room temperature. Another 2 mL opti-MEM was added before mixing with FG cells. After incubation for 6 h, the supernatant was removed, and maintenance medium without penicillin or streptomycin was added. FG cells were collected at 48 h post transfection, and total RNA was extracted using RNAiso (Takara, Dalian, China) and reverse-transcribed into cDNA using a PrimeScript RT-PCR Kit (Takara, Dalian, China). Expression of clathrin, caveolin-1, and dynamin mRNA was detected by qPCR using primers listed in Table 1. Gene expression was calculated according to the 2^-ΔΔCt^ method with the expression of β-actin as an internal control. Protein expressions of clathrin, caveolin-1, and dynamin were detected by IFA. Specifically, 48 h post transfection with siRNAs, FG cells were fixed with 4% paraformaldehyde for 15 min at room temperature and washed three times with PBS. Cells were then incubated with mouse anti-clathrin, anti-caveolin, and anti-dynamin polyclonal antibodies at 37 °C for 1.5 h, followed by incubation with Alexa Fluor 647 conjugated goat anti-mouse Ig at 37 °C for 1 h. FG cells were observed through a fluorescence microscope, and the value of mean fluorescence intensity of all cells in the field was measured using ImageJ software. Three group figures for each specific siRNA was analyzed.

To elucidate the influence of clathrin, caveolin-1, and dynamin knockdown on LCDV infection, FG cells were infected with LCDV particles at 22 °C for 1 h at 48 h post siRNA transfection. The cells were collected at 48 h post LCDV infection, and DNA was extracted using a TIANamp Marine Animals DNA Kit (Qiagen, Hilden, Germany). LCDV copy numbers were detected by qPCR and calculated using a standard curve according to Ct values as described above.

### 4.6. Laser Scanning Confocal Immunofluorescence Microscopy 

To investigate the entry of LCDV mediated by VDAC2 and RACK1 receptors, the co-localization of LCDV with VDAC2 and RACK1, CTB with LCDV, VDAC2 and RACK1, transferrin with LCDV, VDAC2 and RACK1, and caveolin-1 with LCDV, VDAC2 and RACK1, respectively, was detected by laser scanning confocal immunofluorescence microscopy. The co-localization level of merged figures in each group was quantified into scattered blots, values of Pearson’s correlation, and overlap coefficients by using ImageJ software, three merged figures were analyzed and the results of one merged figure were provided for each group. Pearson’s correlation (−1.0–1.0): values less than 0 indicate the absence of co-localization; overlap coefficient (0–1.0): 0.5 indicates 50% of fluorescence in the selected channels was colocalized.

For the co-staining of LCDV with VDAC2 and RACK1, FG cells were grown to a monolayer on circular coverslips in 24-well plates and washed with PBS after removing the supernatant. Cells were then incubated with 100 μL LCDV at a concentration of 4 TCID_50_/mL for 2 h at 22 °C. Following washes with citrate buffer and PBS, cells were fixed with 4% paraformaldehyde for 15 min at room temperature and incubated with mouse anti-LCDV 32 kDa VAP MAbs (1:250) paired with rabbit anti-RACK1 polyclonal antibody (1:500) or rabbit anti-VDAC2 polyclonal antibody (1:500) as primary antibodies at 37 °C for 1.5 h. Thereafter, cells were washed with PBS and incubated with Alexa Fluor 488 conjugated goat anti-mouse Ig (1:500) paired with Alexa Fluor 647 conjugated goat anti-rabbit Ig (1:500) as secondary antibodies at 37 °C for 1 h. After washing with PBS, cell nuclei were stained blue using DAPI at 37 °C for 15 min. Cells were observed under a confocal laser scanning microscope (Leica, Wetzlar, Germany). Rabbit pre-immune serum paired with mouse pre-immune serum instead of primary antibodies served as a negative control.

For the co-localization of CTB with LCDV, VDAC2, and RACK1, as well as transferrin with LCDV, VDAC2, and RACK1, FG cells were incubated with 100 μL LCDV and an equal volume of Alexa Fluor 647 conjugated CTB or transferrin for 2 h at 22 °C. After washing and being fixed as above, FG cells were incubated with rabbit anti-LCDV (1:500), anti-RACK1 (1:500), or anti-VDAC2 (1:500) polyclonal antibody at 37 °C for 1.5 h, respectively, and then incubated with Alexa Fluor 488 conjugated goat anti-rabbit Ig at 37 °C for 1 h. For the co-localization of caveolin with LCDV, VDAC2, and RACK1, FG cells were incubated with mouse anti-caveolin polyclonal antibody (1:100) paired with rabbit anti-LCDV, anti-RACK1, or anti-VDAC2 polyclonal antibody (1:500) as primary antibodies. Subsequently, cells were incubated with Alexa Fluor 647 conjugated goat anti-mouse Ig (1:500) paired with Alexa Fluor 488 conjugated goat anti-rabbit Ig (1:500) as secondary antibodies. After washing with PBS, cells were stained with DAPI to visualize nuclei and observed under a confocal laser scanning microscope. Rabbit pre-immune serum instead of primary antibodies, or rabbit pre-immune serum paired with mouse pre-immune serum instead of primary antibodies, served as a negative control. 

### 4.7. Statistical Analysis 

The statistical analysis of ELISA and qPCR was performed using Prism 7.0 and SPSS 20.0 software (Chicago, IL, USA). All data were expressed as mean ± standard deviation. The qPCR data of each experimental group and control group were firstly analyzed by Levene’s test for homogeneity of variance at a 5% significance level. If variances were considered to be equal, the data would be subjected to a two-sample *t*-test; if not, the Welch’s *t*-test would be performed. Differences were considered statistically significant when *p* < 0.05.

## Figures and Tables

**Table 1:** Primers, siRNAs, and peptides used in this study.

Primer/siRNA/Peptide	Primer Sequence (5′–3′)	Use
qCaveolin-F	GGACCCCAAGCACATAAACG	RT-PCR (caveolin mRNA expression)
qCaveolin-R qClathrin-F qClathrin-R qDynamin-F qDynamin-R beta-actin-F beta-actin-R	GGACAGGATGGCGAAGAAGAT CTCGGGTATCATTGGGGTCA GACGGATGGTGTCTGGGGTA ACGCCCTGTCTCAAATGTATCG AAGCCTATGAAGTCCTCGTGGTT CACTGTGCCCATCTACGAG CCATCTCCTGCTCGAAGTC	RT-PCR (caveolin mRNA expression) RT-PCR (clathrin mRNA expression) RT-PCR (clathrin mRNA expression) RT-PCR (dynamin mRNA expression) RT-PCR (dynamin mRNA expression) RT-PCR (internal control) RT-PCR (internal control)
LCDV-038F	TCTTGTTCAGCATTTACTTCTCGGC	RT-PCR (LCDV copy number)
LCDV-038R	TCTTCTCCTTTAGATGATTTCCC	RT-PCR (LCDV copy number)
Clathrin-siRNA Caveolin-siRNA	GCAACCAAAUGUUCACCAATT CCUUCACCGUCACCAAGUATT	RNA interference assay RNA interference assay
Dynamin-siRNA NC-siRNA	GCCCGUAGACAUUGAGCAUTT UUCUCCGAACGUGUCACGUTT	RNA interference assay RNA interference assay
Clathrin peptide	MADPNTPIRRPISAC	Production of mouse polyclonal antibody against clathrin
Caveolin-1 peptide	NIYKPNNKDMDNDSC	Production of mouse polyclonal antibody against caveolin-1
Dynamin peptide	CPGVPRRPAPRRNQW	Production of mouse polyclonal antibody against dynamin

For ELISA, 96-well microplates were coated with 20 μg polypeptide per well and incubated overnight at 4 °C. The wells were washed three times with PBST (PBS containing 0.05% Tween-20, *v*/*v*) and then blocked with 4% bovine serum albumin (BSA, Sigma, St. Louis, MO, USA) at 37 °C for 2 h. After washing as above, the polyclonal antibodies diluted from 1:100 to 1:6400 in PBS were added as a primary antibody and AP-conjugated goat-anti-mouse Ig (Sigma, St. Louis, MO, USA) diluted 1:5000 in PBS was used as secondary antibody. After washing with PBST, 100 μL substrate solution (1% diethanolamine, 0.5 mM MgCl_2_, *v*/*v*, pH 9.8) containing 0.1% p-nitrophenyl phosphate (pNPP, Sigma, St. Louis, MO, USA) was added to each well, and absorbance was measured with an automatic ELISA reader (Molecular Devices, Union city, CA, USA) at 405 nm. Mouse pre-immune serum instead of primary antibodies served as negative controls. The experiments were performed in triplicate.

## Supplementary Materials

Supplementary materials can be found at https://www.mdpi.com/1422-0067/21/13/4722/s1.
