# Supplementary material for: Lymphocystis Disease Virus (Iridoviridae) Enters Flounder (Paralichthys olivaceus) Gill Cells via a Caveolae-Mediated Endocytosis Mechanism Facilitated by Viral Receptors
Source: Int J Mol Sci. 2020 Jul 2;21(13):4722. doi: 10.3390/ijms21134722 (PMC7370161; doi:10.3390/ijms21134722)
Supplement: Supplementary file 1 [file ijms-21-04722-s001.pdf]

International Journal of
*Molecular Sciences*

**Supplementary materials**

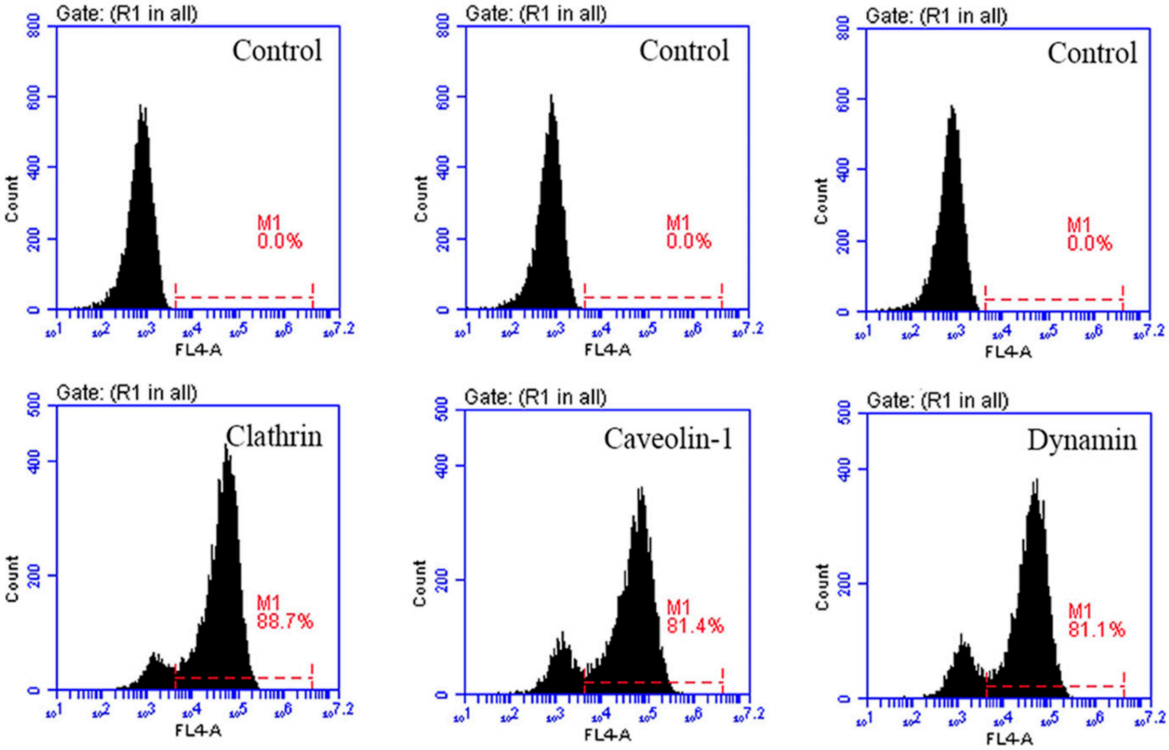


**Figure S1.** Specificity analysis of mouse anti-clathrin, anti-caveolin-1, and anti-dynamin polyclonal antibodies. Clathrin-, caveolin-1-, and dynamin-positive FG cells determined by FACS analysis.

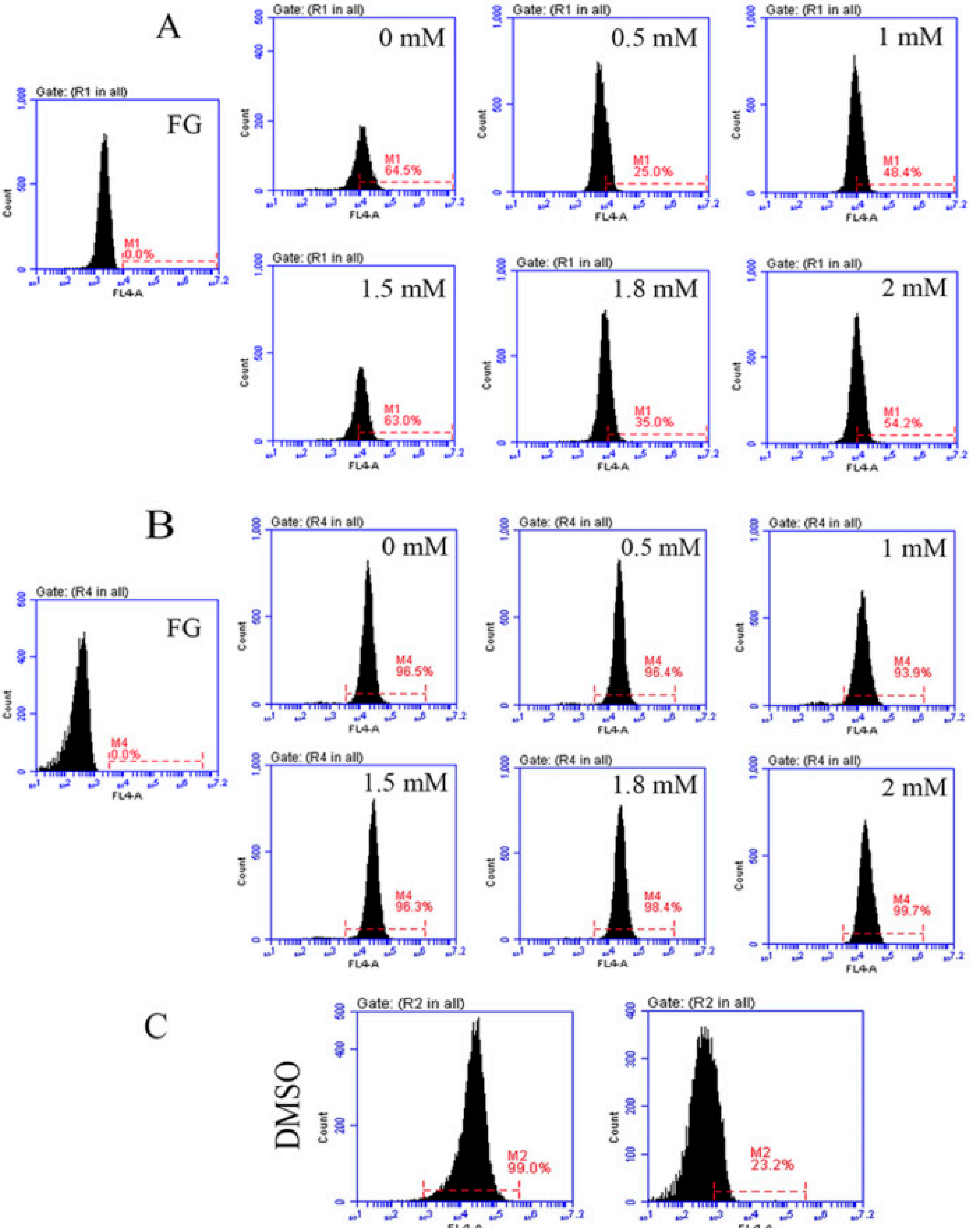


**Figure S2.** Cholesterol is required for LCDV entry. (**A**) FG cells were pre-incubated with MβCD at 22 °C for 1 h, then incubated with LCDV for another 1 h in the continued presence of reagent. FACS analysis was performed with anti-LCDV 32 kDa VAP polyclonal antibody to visualize LCDV-positive cells. (**B**) FG cells were infected with LCDV for 1 h and then treated with different concentrations of MβCD at 22 °C for 1 h. LCDV-positive cells were detected by FACS. (**C**) FG cells were infected with LCDV for 1 h and then treated with different concentrations of MβCD at 22 °C for 1 h. LCDV-positive cells were detected by FACS. FG cells treated with DMSO instead of MβCD prior to and post LCDV infection served as negative controls. FG cells without LCDV infection and reagents treatment served as blank controls.

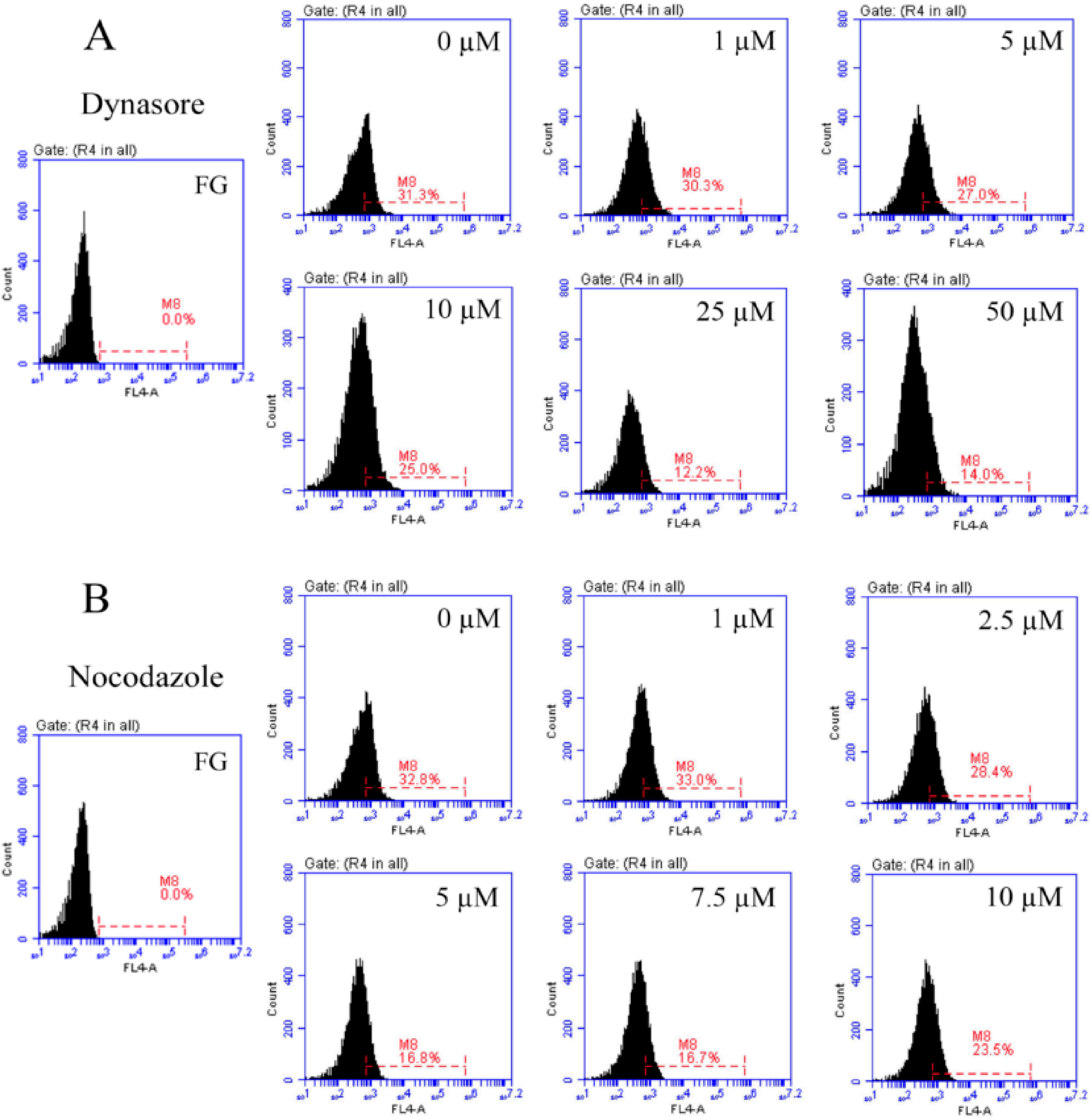


**Figure S3.** LCDV entry depends on dynamin and microtubules. FG cells were pre-incubated with dynasore (**A**) and nocodazole (**B**), and then infected with LCDV. LCDV-positive cells were detected by FACS. FG cells pre-incubated with DMSO instead of dynasore or nocodazole served as negative controls. FG cells without LCDV infection and reagents treatment served as blank controls.

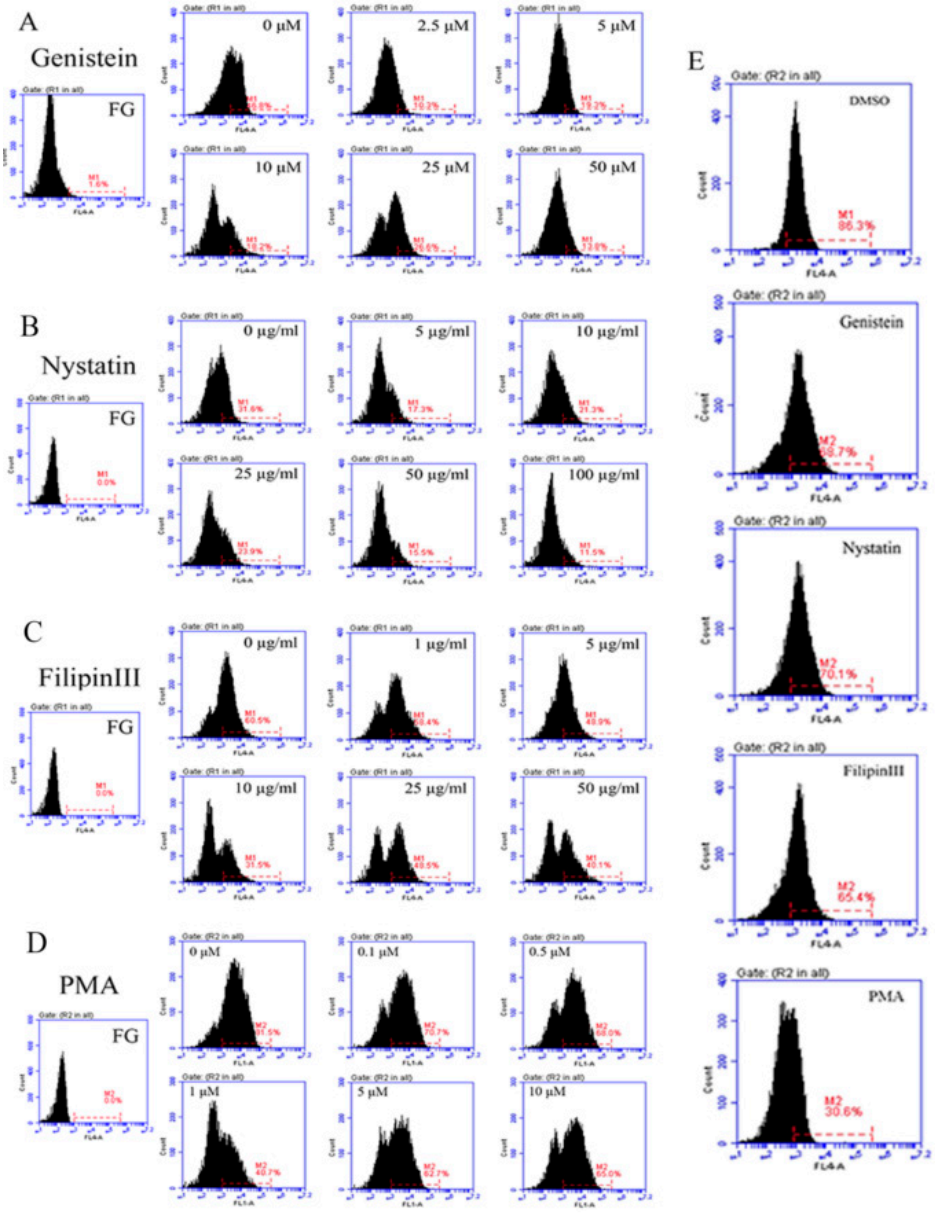


**Figure S4.** LCDV enters cells via caveola-dependent endocytosis. FG cells were pre-incubated with genistein (**A**), nystatin (**B**), filipin III (**C**), or PMA (**D**), and then infected with LCDV. LCDV-positive cells were detected by FACS. (E) FG cells were treated with 10 µM genistein, 25 µg/mL nystatin, 10 µg/mL filipin III, or 1 µM PMA, then incubated with Alexa fluor 647 conjugated CTB. CTB-positive cells were detected by FACS. FG cells treated with DMSO instead of genistein, nystatin, filipin III, or PMA served as negative controls. FG cells without LCDV infection and reagents treatment served as blank controls.

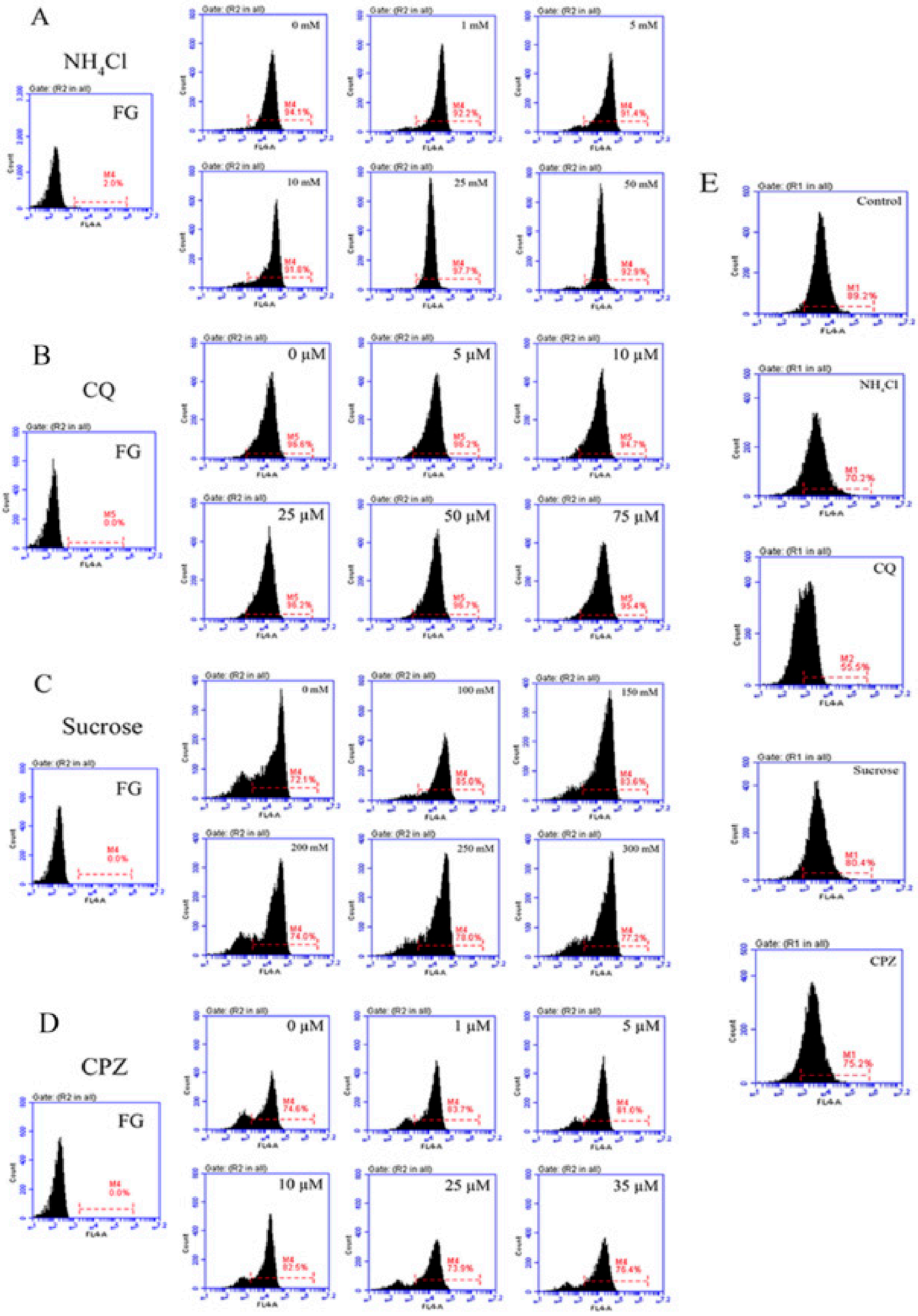


**Figure S5.** LCDV entry is pH- and clathrin-independent. FG cells were pre-incubated with $NH_4Cl$ (**A**), CQ (**B**), sucrose (**C**), or CPZ (**D**), and then infected with LCDV. LCDV-positive cells were detected by FACS. FG cells were treated with 10 mM $NH_4Cl$, 25 μM CQ, 200 mM sucrose, or 10 μM CPZ, then incubated with Alexa fluor 647 conjugated transferrin. Transferrin-positive cells were detected by FACS (E). FG cells treated with distilled water instead of $NH_4Cl$, CQ, sucrose or CPZ served as negative controls. FG cells without LCDV infection and reagents treatment served as blank controls.

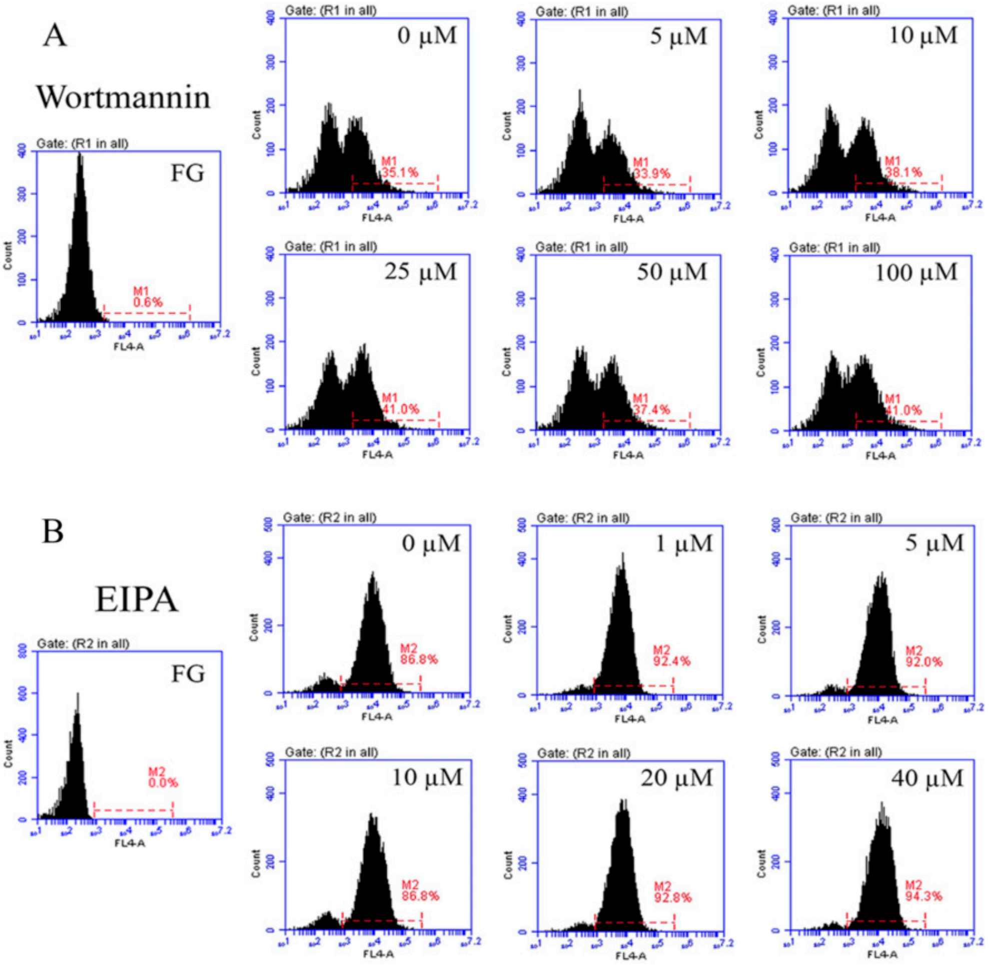


**Figure S6.** LCDV entry is macropinocytosis-independent. FG cells were pre-incubated with wortmannin (**A**) or EIPA (**B**), and then infected with LCDV. LCDV-positive FG cells were detected by FACS. FG cells treated with DMSO instead of wortmannin or EIPA served as negative controls. FG cells without LCDV infection and reagent treatment served as blank controls.

**Table S1.** Statistical analysis results for each of the tests performed.

| Reagents | Concentration | Levene's Test | | T Test / Welch's T-Test | | |
|---|---|---|---|---|---|---|
| | | F (0.05, 1, 4) | Sig. | t value | df value | *p* value |
| MßCD | 0.5 mM | 9.756 | 0.035 | 10.572 | 2.152 | 0.007 |
| | 1 mM | 7.349 | 0.053 | 7.279 | 4 | 0.002 |
| | 1.5 mM | 10.911 | 0.03 | 8.481 | 2.097 | 0.012 |
| | 1.8 mM | 13.882 | 0.02 | 9.941 | 2.013 | 0.010 |
| | 2 mM | 12.556 | 0.024 | 8.997 | 2.043 | 0.011 |
| | 0 mM | / | / | / | / | / |
| Dynasore | 1 μM | 0.18 | 0.693 | 5.395 | 4 | 0.006 |
| | 5 μM | 1.152 | 0.344 | 12.754 | 4 | 0.000 |
| | 10 μM | 0.085 | 0.786 | 8.722 | 4 | 0.001 |
| | 25 μM | 0.126 | 0.741 | 6.575 | 4 | 0.003 |
| | 50 μM | 0.301 | 0.613 | 7.565 | 4 | 0.002 |
| | 0 μM | / | / | / | / | / |
| Nocodazole | 1 μM | 0.008 | 0.933 | 24.611 | 4 | 0.000 |
| | 2.5 μM | 0.38 | 0.571 | 18.936 | 4 | 0.000 |
| | 5 μM | 1.401 | 0.302 | 12.355 | 4 | 0.000 |
| | 7.5 μM | 0.432 | 0.547 | 50.131 | 4 | 0.000 |
| | 10 μM | 0.818 | 0.417 | 40.115 | 4 | 0.000 |
| | 0 μM | / | / | / | / | / |
| Genistein | 2.5 μM | 8.522 | 0.043 | 49.55 | 2.151 | 0.000 |
| | 5 μM | 5.991 | 0.071 | 18.105 | 4 | 0.000 |
| | 10 μM | 5.379 | 0.081 | 12.081 | 4 | 0.000 |
| | 25 μM | 0.556 | 0.497 | 10.025 | 4 | 0.001 |
| | 50 μM | 3.916 | 0.119 | 3.149 | 4 | 0.035 |
| | 0 μM | / | / | / | / | / |
| Nystatin | 5 μg/ml | 0.125 | 0.741 | 48.467 | 4 | 0.000 |
| | 10 μg/ml | 0.094 | 0.774 | 55.823 | 4 | 0.000 |
| | 25 μg/ml | 11.577 | 0.027 | 89.656 | 2.023 | 0.000 |
| | 50 μg/ml | 0.391 | 0.566 | 64.048 | 4 | 0.000 |
| | 100 μg/ml | 2.558 | 0.185 | 66.432 | 4 | 0.000 |
| | 0 μg/ml | / | / | / | / | / |
| Filipin III | 1 μg/ml | 0.337 | 0.593 | 5.804 | 4 | 0.004 |
| | 5 μg/ml | 0.158 | 0.711 | 4.919 | 4 | 0.008 |
| | 10 μg/ml | 0.416 | 0.554 | 5.89 | 4 | 0.004 |
| | 25 μg/ml | 0.041 | 0.849 | 5.039 | 4 | 0.007 |
| | 50 μg/ml | 0.903 | 0.396 | 5.001 | 4 | 0.007 |
| | 0 μg/ml | / | / | / | / | / |
| PMA | 0.1 μM | 6.876 | 0.059 | 4.926 | 4 | 0.008 |
| | 0.5 μM | 2.64 | 0.18 | 2.791 | 4 | 0.049 |
| | 1 μM | 0.146 | 0.722 | 7.797 | 4 | 0.001 |
| | 5 μM | 2.728 | 0.174 | 4.634 | 4 | 0.010 |
| | 10 μM | 10.398 | 0.032 | 11.221 | 2.08 | 0.007 |
| | 0 μM | / | / | / | / | / |
